# Distinguishing multiple roles of T cell and macrophage involvement in determining lymph node fates during *Mycobacterium tuberculosis* infection

**DOI:** 10.1371/journal.pcbi.1013033

**Published:** 2025-05-07

**Authors:** Kathryn C. Krupinsky, Christian T. Michael, Pariksheet Nanda, Joshua T. Mattila, Denise Kirschner

**Affiliations:** 1 Department of Microbiology and Immunology, University of Michigan - Michigan Medicine, Ann Arbor, Michigan, United States of America; 2 Department of Infectious Disease and Microbiology, Graduate School of Public Health, University of Pittsburgh, Pittsburgh, Pennsylvania, United States of America; University of Tennessee Health Science Center College of Medicine Memphis, UNITED STATES OF AMERICA

## Abstract

Tuberculosis (TB) is a disease of major public health concern with an estimated one-fourth of the world currently infected with *M. tuberculosis* (Mtb) bacilli. Mtb infection occurs after inhalation of Mtb, following which, highly structured immune structures called granulomas form within lungs to immunologically restrain and physically constrain spread of infection. Most lung granulomas are successful at controlling or even eliminating their bacterial loads, but others fail to control infection and promote disease. Granulomas also form within lung-draining lymph nodes (LNs), variably affecting immune function. Both lung and LN granulomas vary widely in ability to control infection, even within a single host, with outcomes ranging from bacterial clearance to uncontrolled bacterial growth. While lung granulomas are well-studied, data on LN granulomas are scarce; it is unknown what mechanisms drive LN Mtb infection progression and variability in severity. Recent data suggest that LN granulomas are niches for bacterial replication and can reduce control over lung infection. To identify mechanisms driving LN Mtb infection, we developed a multi-scale compartmental model that includes multiple lung-draining LNs, blood. We calibrated to data from a nonhuman primate TB model (one of the only models that parallels human TB infection). Our model predicts temporal trajectories for LN macrophage, T-cell, and Mtb populations during simulated Mtb infection. We also predict a clinically measurable infection feature from PET/CT imaging, FDG avidity. Using uncertainty and sensitivity analysis methods, we identify key mechanisms driving LN granuloma fate, T-cell efflux rates from LNs, and a role for LNs in pulmonary infection control.

## Author summary

Despite a strong prevalence during pulmonary *Mycobacterium tuberculosis* (Mtb) infection, lymph node (LN) Mtb infection is not well understood. There exists an incomplete understanding of how infection in LNs, a host’s primary site of pathogen-specific immune cell generation, impacts overall host immune responses. To better comprehend LN Mtb infection progression and its role within pulmonary Mtb infection, we developed a multi-scale mathematical compartmental model that captures key infection mechanisms calibrated to data from a nonhuman primate TB model (one of the only models that parallels human TB infection). To our knowledge, this is a first-of-its-kind model for LN Mtb infection and analysis of this model identifies new avenues of research regarding long-term control and treatment of Mtb infection. We performed analyses on this model to determine bacterial and host factors that drive LN infection outcomes and how LN Mtb infection impacts host LNs ability to aid in controlling pulmonary Mtb infection.

## Background

Tuberculosis (TB) is an ancient disease with recorded human cases as early as 1700 BCE [1]. Since its emergence within the human population, many techniques have been used to understand the disease. Predating the discovery of the causative bacterium, epidemiological techniques were used to derive knowledge about the optimal treatment of disease and factors impacting its spread within a population [2]. Following discovery in 1882 of the causative agent, *Mycobacterium tuberculosis* (Mtb) [2], extensive *in vitro* and *in vivo* experiments have been conducted to understand how the pathogen causes disease. More recently, *in silico* modeling of Mtb’s interaction with the host has led to discoveries about optimal drug regimens [3,4] and factors leading to different disease states [5–7]. We will detail more about the notable discoveries in the field of Mtb research within the Background section.

It is currently estimated that a quarter of the world has been exposed to or is currently infected with *Mycobacterium tuberculosis* (Mtb), the causative agent of TB. Mtb is transmitted through the respiratory route and infection leads to hosts developing granulomas within their lungs (pulmonary infection). Pulmonary granulomas are hallmark structures of Mtb infection and a primary focus of research. Multiple granulomas form in response to infection [8]. These highly-structured immune complexes isolate Mtb and, if successful, control Mtb infection. Multiple granulomas form within lungs of infected hosts [8] and each pulmonary granuloma’s ability to control its Mtb burdens is highly variable [8,9]. The human immune system has potent tools for controlling Mtb infection and approximately 80–90% of those infected never develop symptomatic disease, instead progressing to asymptomatic (latent) infections [10,11]. Individuals with latent TB infection (LTBI) typically do not know that they are harboring Mtb, complicating infection identification and treatment [12]. Furthermore, individuals with LTBI may lose control over their infections over their lifetime leading to reactivation of active TB. Active TB disease is highly contagious and is a serious disease that is fatal in 10–20% of patients if left untreated [10,11].

While lung granulomas are the focus of much investigation in TB, lymph node (LN) infection is an important aspect of this disease that receives less attention. During Mtb infection, multiple lung-draining (thoracic) LNs respond to antigen presentation via dendritic cells arriving from lung granulomas. In response, LNs supply CD4 + and CD8 + T cells to lung granulomas to participate in an active immune response. Data suggest that immune cell activation accomplished by CD4 + T cells is essential for effective infection containment during Mtb infection [13]. LNs are critical for developing immune responses that facilitate protection against disease, including infection with Mtb throughout the body. Alarmingly, LNs can become diseased: lung-draining (thoracic) LNs are among the most common sites of extrapulmonary TB potentially impacting immune functionality [14].

**Fig 1:**
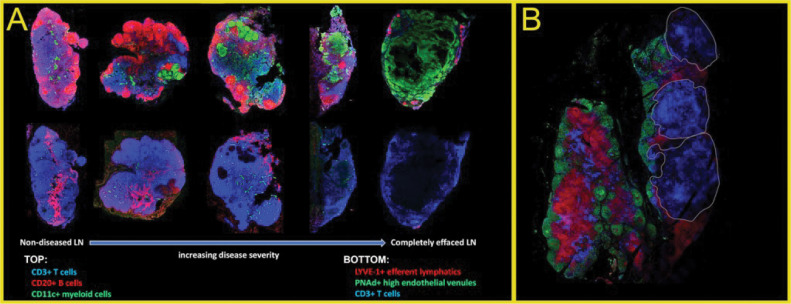
Spectrum and heterogeneity of lymph node (LN) condition during Mtb infection. (A) Five sections of LNs taken from Mtb-infected nonhuman primates (NHPs), arranged by increasing infection severity. The columns are adjacent sections from the same LN stained with different panels of antibodies. The left-most panels present (top) normal arrangements of cell populations including CD3 + T cells, CD20 + B cells, and CD11c+ myeloid cells (macrophages and dendritic cells); and (bottom) normal vasculature architecture. LNs with increasingly severe disease are shown from left to right, culminating in a LN that is completely effaced by granuloma-associated macrophages (right). (B) LNs from the same animal, or even adjacent segments of a single LN, can have substantially different levels of disease. In this LN, B cells (green), T cells (red), and CD11c + DCs and macrophages (blue) are shown in a non-diseased (left) and effaced (right) segments from the same LN.

Identifying mechanisms driving dissemination from lungs to LNs is an active area of research, and many models by which this can happen have been developed [15]. Early radiograph-based studies identified lymphadenopathy in conjunction with pulmonary granulomas–together, called Ghon complexes–and showed that the presence of LN infection during pulmonary infection is common and may be important [16]. More recently, non-human primate (NHP) studies show that LN infection is heterogenous in presentation [17]. These presentations range from LNs with no notable granuloma formation to LNs with severe infection, where granuloma formation completely effaces and destroys normal LN architecture; the full range of this disease can sometimes occur within a single individual [17] (**Fig 1**).

As LNs are primary sites of T-cell priming and expansion, LN granulomas are constantly surrounded by circulating and clonally expanding immune cells [18]. Consequently, LN and lung granulomas have distinct cellular compositions and likely utilize distinct mechanisms to control infection [17]. Presently, it is not clear how LN infection affects T-cell priming or how T-cell priming affects LN Mtb infection progression. Given that pulmonary disease can be controlled by engagement with the adaptive immune system [19] and studies have shown reactivation following decline of CD4 + T-cell populations [20], one hypothesis for eventual reactivation of pulmonary infection is a decline in LN function. This happens in other diseases such as in cancer, where existence of cancer cells within LNs promotes tumor-specific immune tolerance of metastatic processes in distant tissues [21]. It is unclear whether similar mechanisms are at play during Mtb infection until we better understand basic LN function during Mtb infection.

Available *in vivo* models to study Mtb infection within LNs are scarce. Mice do not exhibit granuloma formation and have only a single lung-draining LN [22]. Guinea pig models exhibit both pulmonary and extrapulmonary disease following aerosol exposure but lack complexity seen within human disease [22,23]. Given invasiveness of LN-specific studies, data on human LNs are typically provided post-autopsy or, in some cases, PET/CT scans can provide low-resolution temporal information. Non-human primates (NHP) are an important *in vivo* model of LN-Mtb infection, providing time-series data on inflammation and progression via PET/CT imaging and detailed immunologic and histologic data post-necropsy [24]. NHP studies (particularly with *Cynomolgus macaques*) capture a full range of LTBI and active TB disease states, as well as intra-host heterogeneity as observed in humans [25]. These data can be coupled with known cell-scale mechanisms to develop *in silico* models.

Mathematical and computational models can assist with key analysis to better understand infection dynamics with applications ranging from basic mechanisms of development [26–28] to impact on epidemiological scales [29,30]. The umbrella term of mathematical modeling encompasses multiple approaches for mathematical and computational representations of a target system [31]. For systems with multiple physiological compartments or scales, Ordinary differential equations (ODEs) are a good first approach. For capturing mechanisms assumed to be influenced by tissue geometry and/or rarer events, many modelers elect to use either partial differential equations or agent-based models (ABMs) [4,32,33]. Multi-scale models (MSMs) provide an *in silico* decision-making tool to help identify promising future experimental targets across physiological scales. For example, MSMs integrate known cell-scale mechanisms with experimental data to predict infection outcomes that read out at high scale levels [31]. A recent MSM mechanistically linked from molecular to whole-host scales and recapitulated a fully immunocompetent CD4 + T-cell priming response to antigen [34]. For our studies, special consideration is required to model the interplay between Mtb and host immune cells in the context of granuloma formation occurring between physiological compartments. In our previous work we accomplish this by using MSMs to study pulmonary TB at multiple biological scales ranging from molecular-to-tissue scales [4,33,35–38] and cell-to-whole-host scales [39,40].

Thus, for a first iteration exploring LN granuloma formation and the role of lung-draining LNs infection during pulmonary Mtb infection, we developed an ODE-based non-linear, compartmental mathematical model that captures phenomena occurring in different physiological compartments of lungs, LNs and blood. This compartmental model elucidates drivers of a wide range of infection outcomes seen in LNs during Mtb infection. Further, we use our model to identify mechanisms that predict LN bacterial load, granuloma metabolic activity, and effector T-cell efflux from LNs. By doing this, we uncovered immune factors leading to LN granuloma progression and describe how LN granulomas likely contribute to pulmonary infection.

## Results

In this work, we explore the role of LN Mtb infection and LN granuloma formation during Mtb infection and the connection of these compartments via cells traveling through blood. We use a system of ODEs that represent populations of Mtb-specific and Mtb-non-specific T cells, macrophages, and mycobacteria to identify factors that predict LN granuloma fate. Briefly, we developed a system of 21 ODEs for each of 5 LNs and 16 ODEs for cells within blood. These ODEs detail LN granuloma formation and host-pathogen interactions, antigen presentation and clonal expansion processes within LNs, particularly in response to different states of pulmonary infection (see **Methods**, Sections 3–6 for additional details of modeled processes). While experimentally derived data from a NHP system is only available for 200 days post infection (dpi), we extend our simulations to 480. Our model is able to match those first 200 days and then predict the next 280 days, representing a year and a half of Mtb infection. Through longer time simulations, our extended time frame for the model outcome predicts outside experimental data availability and is significant as (1) the known long-term importance of Mtb infection in the lungs and LNs and (2) we predict interesting disease outcome findings that only present themselves in the post-200-dpi period. Critically, we distinguish between individual virtual hosts via parameterization of each ODE from within a calibrated range (see **Methods**, Section 8). That is, each virtual host has distinctly-parameterized ODEs for both its blood compartment and each of 5 LN compartments, allowing for intra-host heterogeneity.

**Fig 2:**
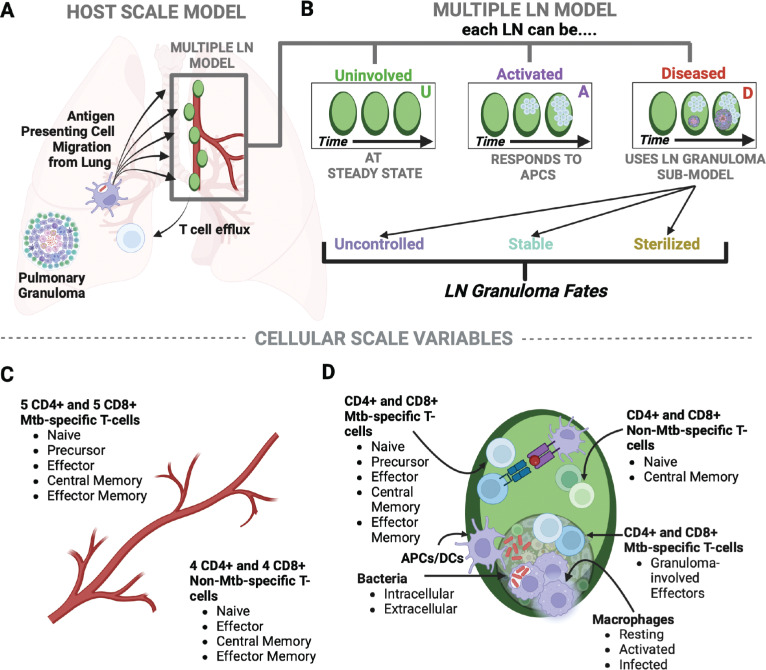
Multi-scale development of multiple LN and LN granuloma sub-models. Our model represents three major scales of study: whole host (lung, LNs and blood) (A), individual LN scale (tissue) (B) and cellular scale (C-D). Immune cell types tracked within each LN and blood, respectively, are listed including macrophages and T cells of different subtypes. Created in BioRender. Krupinsky, K. (2025) https://BioRender.com/h16o401.

Multiscale models capture dynamics of a biological system over different physiological scales and between physiological compartments [41]. In our model, we explicitly represent whole-host scale (lung, LNs and blood) (Fig 2A), individual LN (tissue scale) (Fig 2B), and cellular scales (Fig 2C and 2D). We also represent measurable outcomes for both individual and total LN granulomas (total bacterial burden, e.g.,), whole-LN scale (effacement, e.g.,), and whole-host scale (e.g., T-cell efflux/ net immune response) (Table 1).

**Table 1:** *Summary of multi-scale model outcome metrics.* To predict host fates, we measure multiple outcomes from each LN and, for diseased LNs, each LN granuloma. These include predictions of total bacterial burden (CFU), time-to-sterilization, and effacement.

LN-Granuloma (cell/tissue scale)	Whole-LN (Tissue Scale)	Whole-Host Scale
Total bacterial burden (CFU) Total macrophage count Time-to-sterilization	Predicted effacement (diseased) Mtb-specific and total T-cell count Predicted FDG avidity	T-cell efflux

**Fig 3:**
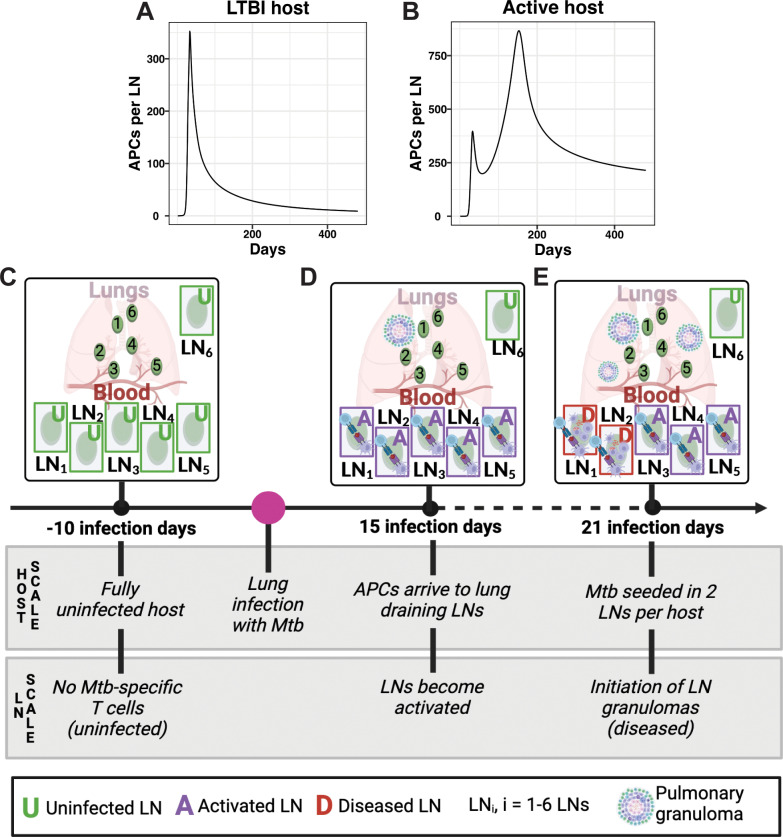
Experimental design for virtual infection: Virtual hosts defined and measured over time using a multi-scale model (MSM). (A, B) Trajectory of antigen presenting cells (APCs) delineating the difference between virtual hosts representing (A) LTBI and (B) active pulmonary infection. These trajectories, generated from our *HostSim* model of pulmonary infection [39], capture two major motifs of how APCs are sent from lungs to LNs in response to multiple lung granulomas (see S1 Fig). Details of different compartments for the MSM are in Fig 2. (C) Prior to pulmonary infection, multiple virtual lymph nodes within each uninfected host maintain a stable, steady state population of immune cells. (D) Following a simulated pulmonary infection with Mtb at day 1, individual LNs become activated when APCs carrying Mtb antigen from the lungs enter the LN by day 15 and antigen presentation induces clonal expansion of T cells. (E) We examine how infection within a LN impacts outcomes by inducing infection within two LNs for each host (i.e., seed them with viable Mtb at day 21); LN granuloma formation follows in those LNs (referred to as diseased). (C-E) were created in BioRender. Krupinsky, K. (2025) https://BioRender.com/m68b077.

For the analyses presented in this study, Panel 2B highlights outcomes for three specific LN fates once seeded with not just antigen, but viable Mtb bacilli: LN granulomas with minimal involvement during infection, LN granulomas controlling infection with stabilizing bacterial growth, and LN granulomas unable to control infection with uncontrolled bacterial growth and destruction of LN architecture (effacement) (Fig 2B). In our model, we define these LN fates based on bacterial load (measured in units of CFU, see **Methods**, Section 9.1 for additional details). Additionally, virtual hosts with LTBI versus those with active pulmonary infection have different dynamics within their lung-draining LNs based on different antigen-presenting cell (APC) profiles (Fig 3A and 3B). For hosts with active pulmonary infection, the APC profile is distinctly bi-modal in contrast to the APC profile for LTBI hosts. This is due to active pulmonary hosts having two uncontrolled lung granulomas leading to continual stimulation and sending of APCs to the LNs (see **Methods**, Section 4 for additional details). We specifically distinguish outcomes between these two pulmonary infection profiles throughout (most of the results comparing active cases are presented in the S1 Text).

***LN model calibration captures key dynamics, proportions of disease severity, and granuloma characteristics.*** Fig 3C-E outlines our experimental design for the LN infection protocol. Prior to infection there are no Mtb within virtual hosts (LNs are uninfected). APCs traffic to LNs from lungs bringing antigen (LNs become activated) and finally LN granulomas form when seeded with live Mtb (LNs become diseased). We use our model to investigate the relative roles of T cells and macrophages driving dynamics of this system. To this end, we first seek to validate our model’s ability to produce trends of T-cells similar to published datasets under multiple infection conditions.

It is known that in the absence of host infection, there is a steady-state level of T cells flowing daily through LNs into blood within the human body and that all T cells travelling through LNs sample for their antigen match [42]*.* Therefore*,* we first ensure that a negative control healthy case follows biological data and dynamics (S3A and S3B Fig). Namely, that T cells are at normal healthy T-cell levels (*steady state*) in the absence of pulmonary infection and that there are an equilibrium level of T-cell numbers circulating between blood and LNs. There are no datasets describing these numbers from experiments or literature in either humans or NHPs, so we estimate their likely sizes (see **Methods**, Section 7.2 for additional details). With 1000 non-diseased, healthy virtual hosts (i.e., virtual hosts with no Mtb in their LNs or lungs and no APC-driven activation of LNs), our model captures estimated cell-population sizes of Mtb-specific immune cells both within LNs (S3A and S3B Fig) and in blood (S2A Fig).

**Fig 4:**
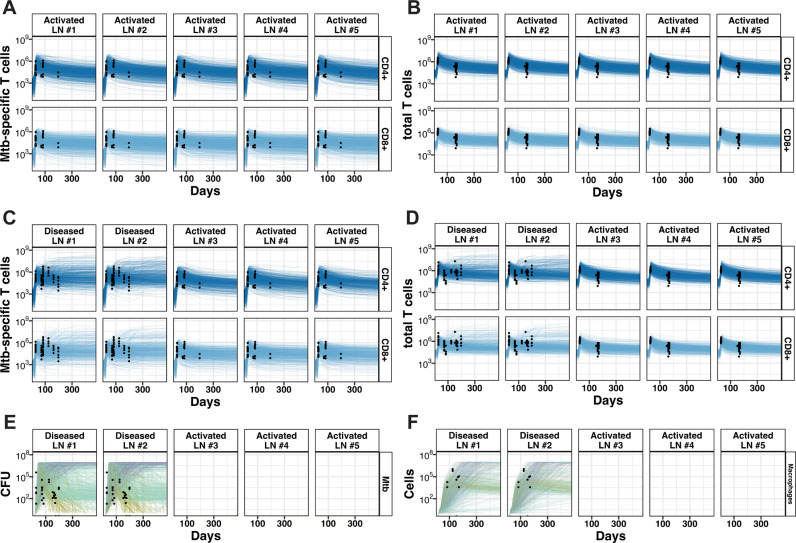
Evolution of immune cell population dynamics in activated and diseased cases within Multiple-LNs for 1000 virtual LTBI hosts. Our model is calibrated to capture key dynamics of Mtb-specific T cells (A, C) and total T cells (B, D) within activated (A, B) and diseased (C, D) cases. Activated hosts have five LNs receiving Mtb activated APCs. Diseased hosts have five activated LNs receiving Mtb activated APCs and LN granulomas forming in LN #1 and #2. For diseased LNs, our model captures the dynamics of LN bacterial load (E) and macrophages (F). We simulated 1000 separate virtual hosts for each case, generating a distinct trajectory for each of their LNs based on their parameterization. Lines in each plot show cell populations from the indicated LN within one host. For LN bacterial load (E) and macrophages (F), lines are colored by bacterial load trajectory: growing large (purple lines), stabilization (teal lines), and sterilization (yellow lines). Flow cytometry data from individual NHP LNs taken at necropsy are represented by black dots from [13]. Note that lines are truncated on virtual host death (see **Methods**, Section 6).

Recent data from NHP studies during Mtb infection indicate that even if a granuloma does not form within a LN, there are still increases in levels of both CD4 + and CD8 + T cells in response to infection (black dots Fig 4A and 4B) [13]. We define a positive control scenario, wherein virtual hosts have five activated LNs (with APCs presenting Mtb epitopes arriving from the lung), *but where granulomas are not forming within LNs* (no live Mtb present - Fig 3D). *In vivo*, the presence of APCs drives recruitment of T cells into LNs [43,44]. Accordingly, virtual hosts have an influx of naïve T cells into a LN in response to APC counts, in our model peaking at approximately 21 days post infection (see Fig 3A and 3B for virtual APC counts; and **Methods,** Section 2 for details) [39,40]. By simulating 1000 virtual hosts with LTBI, we capture general trends and spread of immune-cell data from NHP LNs (black points in Fig 4A and 4B) and blood (S2B Fig) as expected based on known biological mechanisms influencing these processes. Complete details describing calibration processes can be found in **Methods,** Section 8.

Finally, we consider the case of granulomas forming within 2 of the 5 activated LNs (Fig 3E). From Ganchua et al., an average of 19–50% of lung-draining LNs contained viable Mtb (with potential to form granulomas) [13] while other LNs remained activated but not infected (no viable Mtb, but APCs present). We model such hosts with as having five activated LNs (receiving APCs presenting Mtb epitopes - Fig 3A and 3B), two of which become diseased (i.e., seeded with viable Mtb and forming LN granulomas) starting at day 21 post pulmonary infection. This is identical to our positive control (Fig 3D, 3A and 3B) except that within two LNs we seed viable Mtb to initiate granuloma formation. While it is not currently known how antigen presentation/T-cell clonal expansion is impacted by granuloma formation within LNs, we assume a minimal interaction between the two processes: that LN granulomas may recruit effector T cells to participate in granuloma formation and function, rather than allowing them to efflux from LNs to aid in pulmonary immunity. We observe a deviation in T-cell counts from our positive control case once a granuloma starts to form due to Mtb-specific T-cell proliferation within LN granulomas (Fig 4A vs. 4B). Additionally, both Mtb and macrophage populations increase at the beginning of simulated infection and settle into distinct trajectories as infection progresses (Fig 4E and 4F). For 1000 virtual hosts we observe that both Mtb-specific and total T-cell counts have dynamics that reproduce similar behaviors and spreads as seen in NHP data from LNs (black points in Fig 4C and 4D) [13] and in the blood (S2C Fig).

To determine distinctions between the 1000 virtual patients for hosts with LNs that contain APCs alone and those that have 2 granuloma-forming LNs, we explore both simulated and NHP data dynamics of CFU and macrophages for these different cases. We observe distinct T-cell dynamics for simulated LTBI hosts between all three LN scenarios—i.e., LNs that are uninfected (S3A and S3B Fig), activated (Fig 4A and 4B), or diseased (Fig 4C and 4D). For diseased LNs, LN granuloma fates are not clearly distinguishable by T-cell count dynamics alone. Fig 4E shows trajectories for CFU over a 16-month period, and we observe a clear separation between three outcomes of the trajectories: bacterial levels that are growing large (purple lines), bacterial loads that are stable (teal lines), and bacterial levels that sterilize (yellow lines) (see **Methods**, Section 9.1 for details on classification). As can be noted by the large differences in dynamics of each host within each of these three outcomes of the trajectories, these outcomes are broad and still include host-to-host heterogeneity. We overlay data from the same NHP study [13] for macrophages, showing that these distinct outcomes over a 16-month timeframe are not driven by macrophage counts (Fig 4F). S2D-F, S3C, S3D and S4 Figs shows the active TB case for comparison.

Our model equations represent macrophage and T-cell behaviors within individual LNs, and the biology captured in this model has been curated over years [39,40,45,46]. As shown here, our model has been mechanistically calibrated to reproduce LN datasets of T cells, CFU, and macrophages, suggesting that we can infer the impact of T-cell and macrophage behaviors on LN Mtb infection progression. With this well calibrated model, we next investigate key LN-specific outcomes that are expected to depend on mechanisms related to macrophages and T-cells.

**Fig 5:**
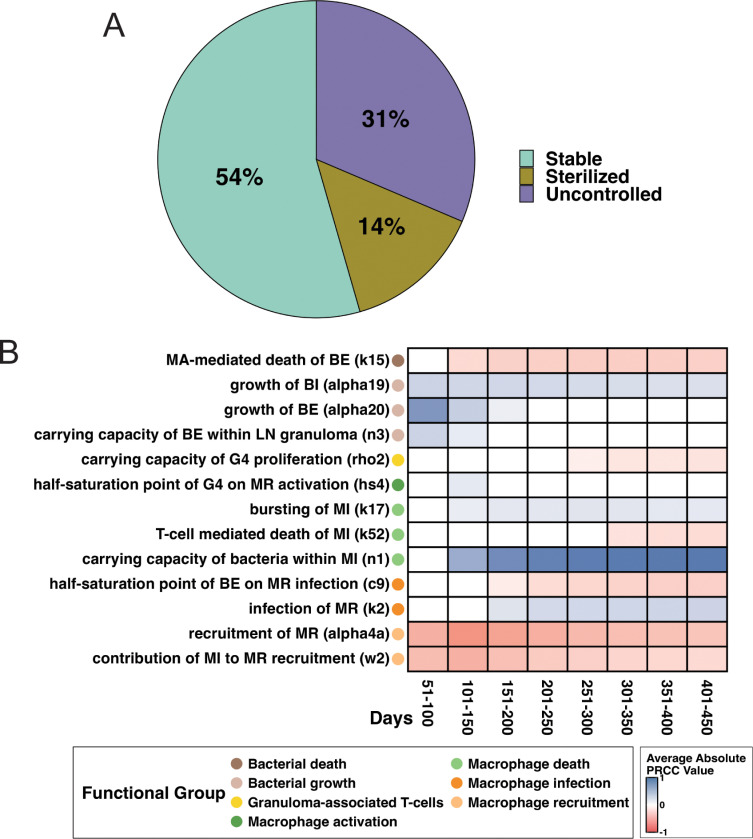
Granuloma bacterial loads are driven by a balance of macrophage infection and activation. *Granulomas are pooled from 1000 LTBI hosts*. (A) Proportion of 2000 virtual LN granulomas by fate: no bacteria present (sterilized), stable bacterial growth (stable), and uncontrolled bacterial growth at 481 days post lung infection (N = 2000). (B) Summary of sensitivity analysis detailing significant parameters driving total bacterial load. PRCCs are binned into 50-day bins for ease of analysis (see Methods). Shading indicates average PRCC value during a time interval *t* (given a parameter is at least significant for 30 days in *t*). White boxes indicate no significant correlation for longer than 30 days in *t*. A blue color indicates a positive correlation, and red color indicates a negative correlation. Significance alpha = 0.01 after Bonferroni correction. Complete model state descriptions (MR, MI, E4, etc.) can be found in Table 2 in Methods and parameter value description found in Tables A, B, and C in S2 Appendix.

***LN bacterial load.*** As observed in NHP infection studies, bacterial loads of individual LNs have unique outcomes [13]. We explore three unique LN granuloma fates: 1) complete bacterial sterilization, 2) granuloma formation and stabilizing Mtb growth, and 3) uncontrolled Mtb growth (Fig 2B). For fates of LNs that are diseased initially, these granuloma fates are defined by bacterial load (CFU) at the end of a simulated infection (day 480) (additional details are found in **Methods,** Section 9.1). We examine 2000 diseased LNs pooled from 1000 virtual hosts for each pulmonary infection scenario (LTBI and active). Among 2000 diseased LNs, a percentage of individual LNs exhibit each of these three bacterial fates (Fig 5A). Surprisingly, within individuals with active lung infection, we see similar percentages of the three granuloma fates (S5A Fig). To determine mechanisms driving these three unique granuloma fates, we performed a sensitivity analysis (see **Methods,** Section 10). Sensitivity analysis explores the influence that each mechanism has on outcomes from our LN model. Partial rank correlation coefficient (PRCC) analysis also ranks the importance of these effects over time. We use this method to identify parameters that most strongly correlate with bacterial load (Fig 5B). From this analysis, we can infer specific biological mechanisms driving bacterial loads within LN granulomas.

**Table 2:** ***State variable symbolic definitions.*** This table contains symbolic and plain text names of state variables and their corresponding descriptions. Plain text names are referenced in Figs 4, 5, 8, and 9. All cells are counted in units of average cell numbers per population.

State Variable	Plain Text Name	Description
$M_{R}$	MR	Resting macrophages
$M_{I}$	MI	Infected macrophages
$M_{A}$	MA	Activated macrophages
$B_{I}$	BI	Intracellular bacteria
$B_{E}$	BE	Extracellular bacteria
$G_{4}$	G4	Granuloma-associated CD4 + T cells
$G_{8}$	G8	Granuloma-associated CD8 + T cells
$N_{4}$	N4	Naïve CD4 + T cells
$P_{4}$	P4	Precursor CD4 + T cells
$E_{4}$	E4	Effector CD4 + T cells
$CM_{4}$	CM4	Central memory CD4 + T cells
$EM_{4}$	EM4	Effector memory CD4 + T cells
$N_{8}$	N8	Naïve CD8 + T cells
$P_{8}$	P8	Precursor CD8 + T cells
$E_{8}$	E8	Effector CD8 + T cells
$CM_{8}$	CM8	Central memory CD8 + T cells
$EM_{8}$	EM8	Effector memory CD8 + T cells

Macrophages and T cells play complex and intertwined roles during Mtb infection [47]. Non-activated macrophages are unable to bind Mtb-containing phagosomes to lysosomes, providing an intracellular replicative niche for Mtb. The relatively slow-growing Mtb replicate inside of infected macrophages, eventually causing them to burst and release bacteria to infect other macrophages [47]. T cells can both induce apoptosis in infected macrophages and activate non-infected macrophages, allowing them to efficiently kill Mtb [48].

Our LN model captures multiple expected interactions between T cells and macrophages (Fig 5B). Negative correlates to total Mtb count include bactericidal activity of activated macrophages (k15) and resting macrophage recruitment rates (alpha4a); positive correlates include Mtb growth rates (alpha19, alpha20). Moreover, we see positive correlation between total LN bacterial load and carrying capacity of Mtb within an infected macrophage (n1). These suggest that Mtb circumvents macrophage carrying capacity restrictions, replicating within a fixed infected macrophage population through macrophage bursting. This has been observed in in both *in vitro* and *in vivo* studies [9,49].

Our sensitivity analysis (Fig 5B) also reveals two distinct temporal effects of macrophage-T cell interactions on total LN bacterial burden: early effects based on T-cell macrophage activation (hs4) and late-stage effects correlated with T-cell mediated macrophage apoptosis (k52). First, we observe a primary role of macrophage activation by granuloma-associated T cells during early infection. This is indicated by a negative correlation between total LN bacterial burden and macrophage activation by granuloma-associated T cells (caused by a positive saturation of the half-saturation point, hs4) between 100–150 days post-infection. From then onward, activated macrophages continue to aid in decreasing total bacterial load within LN granulomas. During late infection (~250 days and beyond), T cells play an important role in directly controlling bacterial levels after a LN granuloma has established (by contrast to indirectly through macrophage activation). A negative correlation between granuloma-associated T-cell proliferation rates (rho2) and bacterial load emerges and, around the same time, T-cell mediated apoptosis of infected macrophages (k52) negatively correlates with total bacteria.

**Fig 6:**
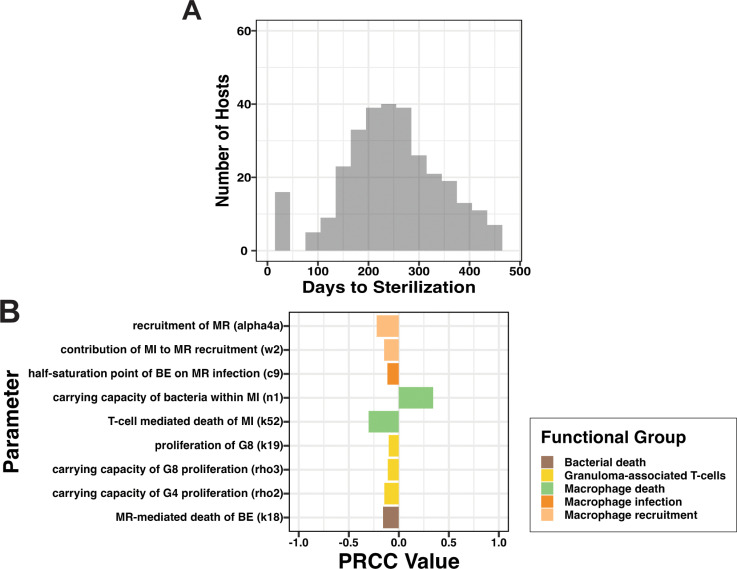
Time-to-sterilization of LN granulomas is driven by macrophage behavior within LTBI hosts. Time to sterilization for a simulated LN is defined as the first-time post-LN-infection that a LN contains less than 0.5 total bacteria, or one day beyond the end of the simulation did not sterilize (see Methods). (A) Time to sterilization among 308 diseased LNs from 1000 simulated hosts that were sterilized within the 480-day simulation period. (B) Significant PRCC correlates between functional groups of parameters and output of interest, namely time-to-sterilization (significance with alpha = 0.01 after Bonferroni correction). Our analysis used 388 individual diseased LNs with granulomas from 1000 simulated hosts. Complete model state descriptions (MR, MI, E4, etc.) can be found in Table 2 and parameter values in Tables A, B, and C in S2 Appendix.

***Time-to-sterilization is lengthened by large extracellular bacterial populations within LNs.*** In Mtb-infected NHPs, even in hosts that have active disease, a substantial proportion of lung granulomas can generate sufficient immune pressure to cause a subset of granulomas to sterilize early [9]. In our LN Mtb infection model, only 14% of LN granulomas sterilize (Fig 5A). This is comparable to frequencies of LN granuloma sterilization that are observed in NHPs at similar time points [13]. To further understand variations in LN granuloma fates based on bacterial load (i.e., sterilization, stabilized growth, or uncontrolled growth) we examine the sterilization case. Among 2000 LNs (from 1000 virtual LTBI hosts), there are 308 diseased virtual LNs that sterilize by the end of simulated infection (481 days post-infection). Sterilization begins in some LNs as early as one-month post-infection while other LNs take as long as 480 days to sterilize (Fig 6A).

To understand specific factors that increase or decrease time-to-sterilization, we perform a sensitivity analysis with time-to-sterilization as our outcome measure (Fig 6B; see **Methods**, Section 10 for details). One important note: time-to-sterilization yields a single value per-sterilizing-granuloma, unlike total bacterial load. This sensitivity analysis indicates which parameters are most predictive of where a granuloma is to fall within the distribution giving rise to Fig 6A. As we expect from our previous section, we see that both recruitment (alpha4a, w2) and proliferation (k19, rho3, rho2) of granuloma-related inflammatory cells (macrophages and granuloma-associated T-cells) correlate with faster time-to-sterilization. This suggests an importance of absolute numbers of immune cells present to determine time-to-sterilization.

The correlates identified in our analysis (Fig 6B) also suggest that LN granuloma time-to-sterilization is worsened by Mtb internalized within macrophages. Macrophage bursting rate (n1) correlates with longer time-to-sterilization, indicating that intracellular Mtb are more difficult to clear, leading to slower sterilization times. This if further supported by a negative correlation between macrophage infection rate carrying capacity (c9) indicating that, as macrophage infection rate is tempered, and as a result extracellular bacterial numbers are allowed to persist, sterilization (i.e., bacterial clearance) is hindered. Relatedly, we find T-cell mediated macrophage death (k52) (which leads to Mtb death or bacteria release into extracellular spaces) reduces time-to-sterilization. Finally, we observe time-to-sterilization shortens with more efficient Mtb-killing by resting macrophage populations (k18) (thereby preventing internalization).

***Predicting LN effacement.*** From our analysis, we observe that LN granuloma fates are determined by numbers of both macrophages and T cells. LN granulomas exist within the context of highly structured and precisely organized LNs. One clinically interesting feature of LNs that contain granulomas is that they typically have some degree of effacement that is induced by granuloma formation [13] (Fig 1A). Effacement presents as structural destruction of LN tissue (necrosis) and narrowing of the anatomic spaces that normally contain the LN’s functional architecture.

Within the NHP dataset, each LN was classified by a pathologist into two categories based on effacement status: greater than (>) 50% effacement and less than (<) 50% effacement. Greater than 50% effacement was based on the observation that approximately more than half of a LN was comprised of structures that were granulomatous material. Those that were less than 50% effacement meant that less than half (or none) of a LN contained granulomatous material. In our study, we use this classification to explore our model outcomes.

**Fig 7:**
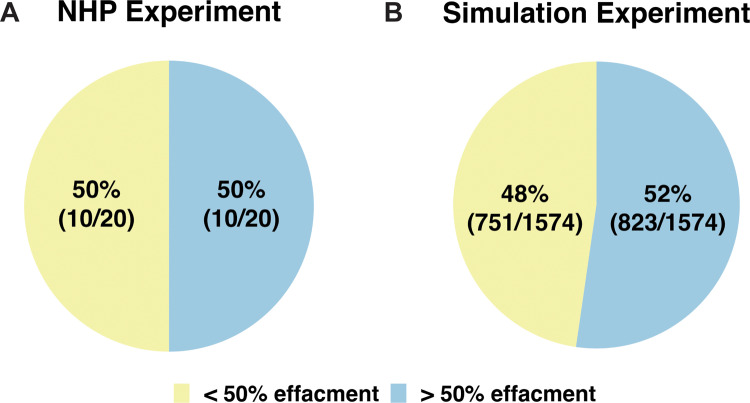
Simulation and experimental comparison: *LN model reproduces NHP LN effacement distributions.* (A) Proportions of experimentally obtained NHP LNs with greater (and less) than 50% effacement at day 201. (B) Proportions of simulated LNs at 201 days post-infection from 2000 virtual LN granulomas from 1000 simulated hosts.

To validate our hypothesis that bacteria loads of LN granulomas drive effacement, we tested whether our model reproduces observed patterns of total LN effacement as observed in NHP LN datasets (from [13]). Total LN effacement directly correlates to LN granuloma size, an outcome calculated based on immune cells and largely driven by total LN granuloma bacterial load (see **Methods**, Section 9.5 for additional details on calculation). To do this, both NHP LNs and simulated infection LNs were divided into two groups: greater than (>) 50% effacement and less than (<) 50% effacement (see **Methods**, Section 9.5 for details). In the analysis, we include all NHP experimental LNs taken before 201 days post-infection; we compare these to simulation LNs from 201 days post-infection. We find that our simulated infection experiment reproduces a similar breakdown of LN effacement (Fig 7). This finding further indicates that our model captures relevant features of T cells, CFU, and macrophages as they relate to LN granuloma formation and maturation. This model validation further increases confidence of our predictions.

**Fig 8:**
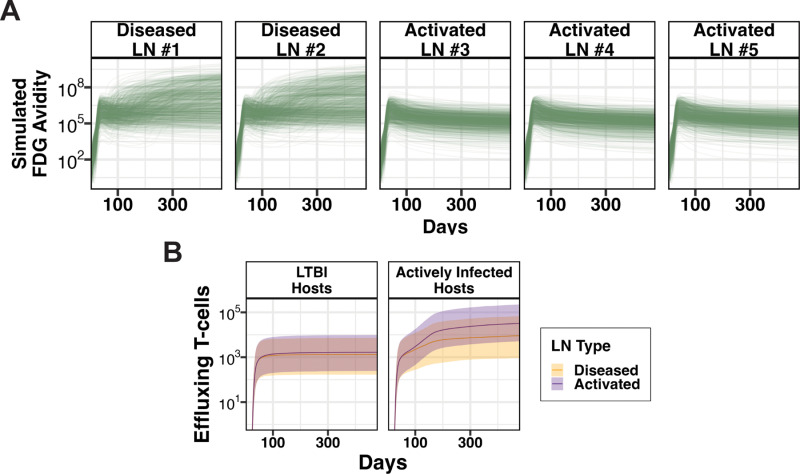
Metrics for tracking infection progression and adaptive immune response. (A) Simulated FDG avidity of each LN over time beginning post-infection (starting at day 1). (B) Number of effluxing effector T cells for hosts with LTBI (left) and active pulmonary infection (right). Solid lines represent the median numbers of effluxing effector T cells from individual LNs (n = 2000 for diseased and n = 3000 for activated, pooled from 1000 virtual hosts). Shaded regions represent interquartile ranges (IQRs).

***Predicting drivers of a clinically-accessible measurement.*** PET/CT images are sometimes available in clinical settings as well as used in experimental NHP studies [50]. One measure taken from these images is FDG avidity. This experimental measurement of relative amount of tagged glucose uptake is the standardized uptake value ratio (SUVR) score. Clinically, PET/CT scans provide information about inflammation occurring in lung granulomas; however, it is incompletely known how this score is impacted by surrounding cells during LN Mtb infection. Despite this, we assume that FDG avidity captures metabolic activity of Mtb infection within humans and NHPs [50,51]. For this study, we simulate a theoretical metric to estimate FDG avidity based on likely immune cell contributors to metabolic activity (see **Methods**, Section 9.3). This metric embeds assumptions about relative metabolic activity by cell type, and so this application of our model is exploratory in nature. That is, we measure relative impact of predicted FDG avidity to provide plausible hypotheses. Towards that goal, we track predicted FDG avidity over time for each LN days post-infection (Fig 8A).

**Fig 9:**
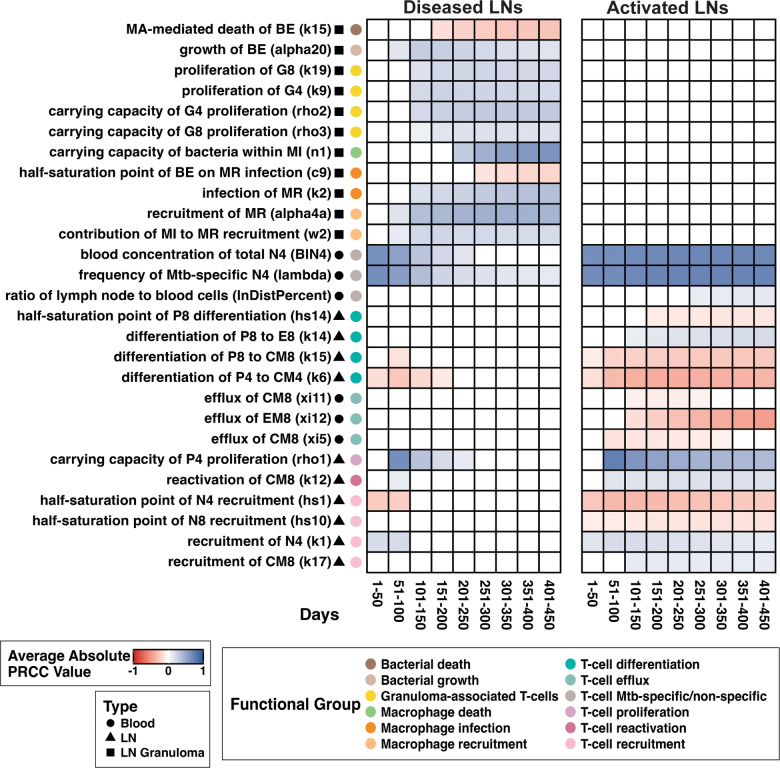
Predicting drivers of FDG avidity within a host with LTBI using sensitivity analysis of simulated FDG avidity. Left panel contains data from 2000 simulated individual diseased LNs and right panel contains data from 3000 individual simulated activated LNs with no granuloma forming. All simulated LNs are taken from the set of 1000 virtual hosts. Shading indicates correlation between parameter and FDG avidity during time interval *t* (given a parameter is at least significant for 30 days in *t*). White boxes indicate that the parameter is not significantly correlated with model outcome at any time points in *t*. A blue color indicates a positive correlation, and red color indicates a negative correlation. Significance alpha = 0.01 after Bonferroni correction. Complete model state descriptions (MR, MI, E4, etc.) can be found in Table 2, and parameters in Tables A, B, and C in S2 Appendix.

To elaborate what drives simulated FDG avidity, we perform a sensitivity analysis (Fig 9). Our sensitivity analysis shows that numbers of Mtb-specific T cells in blood and LN (BlN4, lambda, lnDistPercent) positively correlates with simulated FDG avidity. A greater number of Mtb-specific T cells within a LN prior to infection means more efficient differentiation into cell types that have a higher impact on simulated FDG avidity - i.e., as populations of metabolically active cells within a LN grow, simulated FDG avidity increases. (This case is unlikely unless a host has been previously infected.) We also find that T-cell efflux rates (xi11, xi12, xi5) negatively correlate with simulated FDG avidity, as increases in number of T cells effluxing from a LN lead to fewer T cells present within a LN. Similarly, T-cell recruitment (hs1, hs10, k1, k17) correlates with simulated FDG avidity, reflecting a dependance of simulated FDG avidity on T-cell numbers.

We assume that Mtb-specific effector T cells have the highest weighted contribution to simulated FDG avidity (this is based on activity of activated cells, see **Methods**, Section 9.3 for additional details). Accordingly, we find increases in T-cell priming caused by decreases in the half-saturation parameter (hs14) (an early step along the effector T cell production pathway) and increases in direct differentiation from precursor T cells into effector T cells (k14) correlates with simulated FDG avidity. Thus, rates that directly increase effector production are predictive of increases in simulated FDG avidity. Likewise, T-cell reactivation rates (k12) positively correlate with simulated FDG avidity. Conversely, differentiation rates driving T cells away from effector cell states (k15, k6) negatively correlate with simulated FDG avidity.

For activated LNs, the above-described parameters maintain these correlations throughout the entirety of an infection simulation; however, for diseased LNs, these correlations fade as a LN granuloma matures. Our simulated FDG avidity metric assigns a high weight to granuloma-associated T cells, infected macrophages, and activated macrophages. We find that simulated FDG avidity strongly and positively correlates with increased populations of these cell types (k19, k9, rho2, rho3, c0, k2, alpha4a, w2). We find bacterial growth rates (alpha20) positively correlate with simulated FDG avidity. Bacterial load is not an explicit contributor to our simulated FDG avidity; however, bacterial load increases signals for T-cell recruitment, macrophage activation and infection – all leading to increases in cell types that are highly weighted within our metric. Conversely, increases in bacterial death rates (k15) correlate with decreases in simulated FDG avidity.

**Fig 10:**
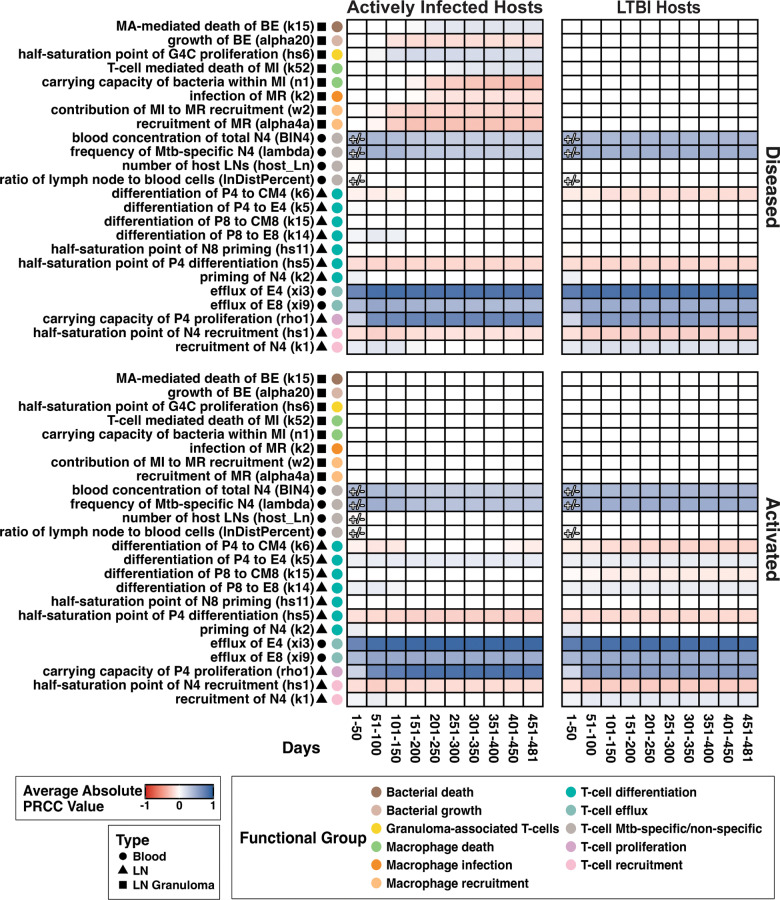
Predicting drivers of Mtb-specific T-cell efflux: comparing LTBI and active hosts using sensitivity analysis of Mtb-specific T-cell efflux. Left panels contain data from 2000 individual simulated diseased LNs and right panels contain data from 3000 individual simulated uninfected/activated LNs. All simulated LNs are from the same set of 1000 virtual hosts. Shading indicates correlation between parameter and FDG avidity during time interval *t* (given a parameter is at least significant for 30 days in *t*). White boxes indicate not significantly correlated at any time points in *t*. A blue color indicates a positive correlation, and red color indicates a negative correlation. Significance alpha = 0.01 after Bonferroni correction. Complete model state descriptions (MR, MI, E4, etc.) can be found in Table 2 and parameters in Tables A, B, and C in S2 Appendix.

***LN granulomas reduce LN ability to aid in fighting pulmonary infection.*** During pulmonary Mtb infection without diseased LNs, the primary role of a LN is to produce effector T cells that traffic back to lungs to aid in controlling pulmonary infection. To do this efficiently, a LN must maintain its highly organized structure that facilitates optimal interaction between APCs and T cells. In the case of diseased LNs, LN structure is physically altered (effaced) by granuloma formation and thus functionality is disrupted. This functionality disruption could occur in two ways: (i) LNs may offer reduced effector T-cell production, or (ii) LNs may produce the same numbers of effector T cells, but some are diverted to engage in anti-Mtb immune responses in LN granulomas instead of effluxing to lungs. Here we assess the potential role of the second mechanism through analysis of our LN model. For virtual LTBI hosts, we see little difference in the number of effector T cells that exit both diseased and activated LNs throughout the course of an infection (Fig 8B, left panel). For virtual hosts with active pulmonary infection, this difference is more pronounced (Fig 8B, right panel). To understand what drives these differences in numbers of effluxing T cells based on LN involvement status and host pulmonary disease status, we performed sensitivity analyses (Fig 10), discussed for the remainder of this section.

The most immediately intuitive drivers of T-cell efflux identified by sensitivity analysis (Fig 10) are consequent to one term in our model’s equations: the number of effector T cells effluxing from a LN (xi3, xi9). This term is proportional to the Mtb-specific effector T-cell population size within that LN. In all cases, we find that the number of Mtb-specific T cells in blood and LN (BlN4, lambda, host_Ln, lnDistPercent) positively correlates with numbers of effluxing effector T cells. Consistent with this, rates of both precursor proliferation within-LN (rho1) and naïve T-cell recruitment-to-LN (hs1, k1) positively correlate with numbers of effluxing effector T cells. During early infection, we observe that naïve T-cell priming (hs11, hs5, k2) has a positive impact on LN effector T-cell counts, indicated by a positive correlation between naïve priming rate and effluxing effector T-cell count.

Similar to our FDG avidity sensitivity analysis (Fig 9), parameters supportive of differentiation of T cells into effector phenotype (k5, k14) positively correlates with numbers of effluxing effector T cells (Fig 10). We also find that parameters that drive differentiation away from effector cells instead into memory cell phenotypes (k6, k15), negatively correlate with numbers of effluxing T cells. This is a general observed trend; however, there are key differences in the duration of time that these trends are significant between each of our LN classifications. Within LNs of LTBI hosts, we find that throughout the entire simulated infection there is a negative correlation between precursor to central memory differentiation rates (k9, k15) and numbers of effluxing T cells. For LNs within hosts that have active pulmonary infection, we find this correlation only during the early stages of infection. This is reflective of the APC profile for hosts with active pulmonary infection that provides stimulation for continued differentiation into effector cells (and not memory cells) throughout the entire simulation period – something that the APC profile of LTBI hosts does not provide (Fig 3A and 3B and **Methods**, Section 2).

Thus, the size of total effector T-cell population within LNs is a major determinant of numbers of effluxing T cells throughout the entire simulated infection with key differences between diseased and active LNs. During both cases, T-cell efflux correlates with multiple parameters associated with increased effector T-cell population sizes (Fig 10). For activated LNs, which lack granuloma formation, this dependency is undisturbed regardless of active pulmonary infection or LTBI; T-cell efflux positively correlates with precursor-to-effector differentiation rates (k5, k14) for the entire infection duration. By contrast, diseased LNs during active pulmonary infection divert effector T cells to within-LN granulomas. This is evident as during early infection, we observe positive correlations between diseased-LN T-cell efflux and effector population size parameters (BlN4, lambda), while these correlations diminish over time as granulomas become established.

As T cells become more effective in aiding control of bacteria, fewer T cells are recruited into LN granulomas, allowing more effectors to efflux. Within diseased LNs of LTBI hosts, we did not find significant correlations between precursor differentiation rates (k6, k5, k14) and numbers of effluxing T cells as in the active case (Fig 10). Within these individuals, T-cell proliferation (hs6) and T-cell mediated infected macrophage death (k52) positively correlate with numbers of effluxing T cells. In hosts that have active pulmonary infection, rates of activated macrophage killing of bacteria (k15) positively correlate with total T-cell efflux. In this case, we also find that bacterial growth rates (alpha20), macrophage recruitment rates (w2, alpha 4a), and macrophage infection rates (k2) each negatively correlate with numbers of effluxing T cells.

## Discussion

LNs are among the most common sites of extrapulmonary TB and may hold the key to understanding how pulmonary infection progresses or participates in reactivation after years of LTBI [17]. Data from LNs during human and primate infection is scarce and usually obtained only at autopsy or necropsy. In this study, we developed a mathematical model that recapitulates individual LN dynamics in both the presence and absence of LN Mtb infection over time. Using our model and available datasets on LNs during Mtb infection in NHPs, we sought to identify mechanistic drivers of LN granuloma outcomes and FDG avidity (a clinical marker of Mtb infection progression). We also aimed to understand specific mechanisms that may lead a LN to inefficiently provide effluxing effector T cells to aid in control of Mtb within an infected lung.

Within the context of Mtb infection, macrophages play a key and complicated role in determining infection progression [52,53]. Macrophages are a replicative niche of Mtb and, when Mtb are taken up by a macrophage, they replicate and evade within-macrophage killing, essentially shielded from antibacterial immune factors [54,55]. However, macrophages can be activated and then are able to directly kill mycobacterial populations. Within our LN model we find that bacterial populations are aided by mechanisms promoting intracellular Mtb survival and harmed by mechanisms promoting mycobacteria within extracellular spaces within a LN granuloma (Fig 5). Similarly, we find that mechanisms promoting intracellular bacterial populations slow time-to-sterilization or fully prevent sterilization, and that mechanisms that lead to externalized Mtb or preventing internalization of Mtb promote earlier LN granuloma sterilization (Fig 6). Specifically, mechanisms supporting macrophage infection/persistence slows time-to-sterilization and mechanisms supporting macrophage death leading to increases extracellular Mtb speeds time-to-sterilization.

Our findings regarding the roles of extracellular/intracellular bacterial populations show that both host and bacterial factors contribute to LN granuloma fates. They also lead to differential infection outcomes, depending on both intensity and combination of host and pathogen mechanisms. This is consistent with a ‘damage-response’ framework of microbial pathogenesis that posits that the specific outcome of a microorganism’s dynamics is a direct result of both host and microorganism mechanisms and their interactions [56,57]. For many mechanisms we represent coarse-grain phenomena for both bacteria and host-factors. For example, our mechanisms are influenced by well-studied features of Mtb biology such as modulation of its microenvironment and replication rates [58]. These features are known to be characteristic of *Mycobacteria*, evidenced by their highly-conserved and low [1–2] rRNA operon copy numbers [59], as compared to other bacterial species, including *E. coli* [60] and *Salmonella enterica,* many of which often have seven or more copies [61]. On the other hand, the importance of host factors is highlighted by conditions like sarcoidosis (a granulomatous condition with no directly identifiable pathogen that is comparable to TB in several ways [62–65]). Other host factors are indicated by the diagnostically-problematic similarities between malignancy and Mtb-infected intestine-adjacent LNs [66], and genetic variability in cytokine expression levels [67]. Together, this suggests that LN granuloma fates are not determined by a single feature (i.e., a virulence factor, as is the case in many microbial infections). Instead, as we have also observed within lung granulomas, it is a balance of host and microbial factors that must be understood to understand infection outcome [68].

Moreover, the importance of this balance of host-pathogen interactions may be intrinsic to transmissible granulomatous conditions. Disruption of this balance may be responsible for the “paradoxical reactions” observed in approximately 20% of LN-diseased TB hosts [69,70]. In those cases, anti-tuberculosis-treated LN granulomas transiently enlarge before eventual resolution [69,70]. This balance is also seen in schistosomiasis, an infectious granuloma-forming disease with LN granulomas in very rare cases [71]. It is similar to TB in that the pathogen maintains a curated level of tissue damage; the resultant inflammation is hypothesized to be important in the pathogen’s transmission cycle [72].

NHP studies show that granuloma fate of one diseased LN does not influence the fate of a different LN in the same host [13]. This is also observed within lungs during Mtb infection where each granuloma is an island with a unique trajectory of dynamics and bacterial load started by a single bacillus [8]. Those findings are organ specific; regarding between organ associations, our model shows that pulmonary infection status does influence LN infection determinants and outcomes via antigen presenting cell information passed from lungs into LNs. Specifically, we find that T cells modulate most measures of LN granuloma control, dependent on direct stimulation from Mtb-antigen-bearing APCs influxing from lungs. Nonetheless, these changes suggest that continued stimulation from APCs when there is an active pulmonary infection as compared to LTBI fundamentally changes the progression and impact of LN granuloma fates.

The role of Mtb-specific T cells versus total T cells has not been deeply explored in primates (humans and NHPs) as tools for identifying specificity is currently limited to tetramers, which are not yet available except for mice and MHCII limited primates like Mauritian cynomolgus macaques [73]. Interestingly, we identified Mtb-specific T cells as a key component driving our FDG metric as well as the metric of time-to-sterilization of LN granulomas suggesting these Mtb-specific cells are worth further exploration. Relatedly, we also find that initial number of Mtb-specific T cells in the blood and LN are key determinants of FDG avidity (Fig 9), suggesting that an individual’s exposure and vaccination history may be important when considering LN FDG avidity. Further, the relevance of pre-existing immunity levels is supported by our observation of levels of Mtb-specific T-cell populations in blood influencing numbers of effluxing effector T cells. However, within most animal studies (including datasets we used to calibrate our model [13]), we assume animals are naïve to Mtb prior to experimental infection (and these animals were not vaccinated). Following first encounter with a microorganism (regardless of infection establishment), a memory immune response is established. With additional data from Mtb-experienced hosts, we can pursue identification of promising mechanisms that could underpin a more targeted mode of Mtb vaccination [74,75]. This Mtb memory may be gained by BCG vaccination (presently administered in much of the world) as is seen in some cases [76]; although how long immune memory lasts is unknown. Further, we estimate total T cells in LNs (including non-specific) using common assumptions from literature (see **Methods**, Section 7.2). Surprisingly, our estimates suggest total T-cell counts in healthy LNs (estimated as 10^6^-10^8^) considerably larger than those measured in antigen-stimulated NHP LNs (measured as 10^4^-10^6^). This inconsistency reveals a need for better characterization of differences between healthy NHP, human, and murine lymphatics.

Like all models, our MSM has limitations that depend on assumptions. First, variety of LN granuloma fates observed may be explained by our choice to only represent three subtypes of macrophages: resting, infected, and activated. This assumption allows us to sufficiently match available NHP data. However, some studies suggest that there may be additional dendritic cells and macrophage subtypes play unique roles in controlling Mtb infection, although these data have not yet been collected within LNs [77,78]. We also do not represent spatial heterogeneity of lymph node granulomas in this work. This is a simplifying (coarse-graining) assumption that would affect representation of processes like drug treatment; however, our validations show that cell scale drivers of LN granuloma fate are within our model’s context of use (see **Methods**, Section 8.3). Additionally, LN necrosis (accumulation of dead cells) is not directly represented in our model. Our data suggests that the organization of LN granulomas differs from lung granulomas that are mostly caseous necrotic in nature. This likely results from immune cells ready at the start of LN granuloma formation, where within lungs it can take anywhere from 3-6 weeks for adaptive immunity to become detectable [19]. In current work we are exploring the role of necrosis in LN granulomas.

Our model is built solely to describe LN dynamics, and this phenomenologically captures dynamic interactions between lungs and LNs during infection. We assume that a function representing APC influx into LNs from lungs (derived from our previously calibrated model of lung infection) represents flow of information to LNs. We also assume that numbers of Mtb-specific T cells within a lung infection is derived solely from the efflux of numbers of effectors T cells leaving a LN. This is a fair assumption to start, however our current work is linking this detailed LN model together with our model that represents lung dynamics with multiple granulomas and blood and lymph, *HostSim* [39]. This will allow us to delve deeper into exact mechanisms of how LN and lung infection affect each other during TB. Our model also assumes that virtual hosts are Mtb-naïve and do not have comorbidities such as human immunodeficiency virus (HIV). Thus, we do not explore the impact of prior TB infection and comorbidities on LN infection progression. We also assume that LN failure does not affect the number of antigen-bearing presenting cells coming from lung into LNs. The impact on dynamic interactions between LN shutdown and pulmonary TB outcomes is currently unknown, and we will explore this in future work.

We independently sample parameters for each virtual LN, an assumption supported by data showing large differences in the ability of individual LNs to control disease. In the future, we could change our sampling method to constrain individual hosts to have more similar LNs, if biological evidence supports this. Additionally, we hope to further develop how we capture mechanisms of LN T-cell population partitioning, where T cells are either granuloma associated or not. This will likely impact development of LN granulomas and LN maturation as well as activation of T cells and T cell efflux to lungs. As no data exists for these values, our current model also does not include any decrease in rates of proliferation and differentiation of T cells in response to LN granuloma formation. This is likely an additional mechanism through which a LN granuloma impacts LN efficiency and will be included in future iterations of the model. In doing this, we will have the capability to determine how LN granulomas impact pulmonary infection control and how they may contribute to the reactivation of pulmonary disease – a pressing question in TB research today and one that may stem directly from LN control.

## Methods

In the present study, we aim to understand the role of granuloma formation within multiple LNs during Mtb infection within LNs. We have previously published a whole host model of TB including lungs, LNs and blood (Section 1). In that model, called *HostSim*, LNs serve solely as a source of T cells to supply the lung granulomas trafficking through blood [39,40]. Here, we expand on this work and describe in detail the development of our multiple LN model with LN granuloma formation capability (Section 2). Our ODE-based model simulates multiple independent LNs (Section 3) that are linked to the virtual host (i.e., whole-host scale model components) through influx of antigen presenting cells (Section 4). A LN granuloma sub-model (Section 5) is embedded within each LN and initiated based on manual input of infected macrophages and intracellular bacterium. To account for whole-host-scale biology, virtual host death is permitted following reaching of a pre-determined total bacterial load threshold (Section 6).

We calibrate and validate our model using NHP datasets, experimental methods used to generate these data are described in Section 7; these data have been previously published [13]. For model calibration, we employ multiple well-validated parameter estimation methodologies (Section 8). We use our model to examine 5 biologically relevant outcomes: LN granuloma bacterial load (Section 9.1), time-to-sterilization (Section 9.2), serial 2-deoxy-2-[18F]-D-deoxyglucose (FDG) avidity (Section 9.3), numbers of effluxing T-cells (Section 9.4), and virtual LN effacement (Section 9.5). To analyze these outcomes, we employ uncertainty and sensitivity analysis using a combination of Latin hypercube sampling and partial rank correlation coefficients (PRCC) (Section 10). We describe model implementation and software in Section 11.

## 1. Model selection and development

Previously, our lab has developed several models to study lymph nodes during infection. First, we developed a novel model of blood and lymph node infection during HIV-1/AIDS infection [79,80]. Next, we adapted that model to study Mtb during Mtb infection [45,81–82]. These models assume that a LN is a “well-mixed” homogeneous compartment and that there is no spatial component to the dynamics, a good approximation for the questions we were asking. We also studied dynamics of T cells and dendritic cells trafficking within LNs using an agent-based model to capture the intricate spatial dynamics of individual cells locating each other within LNs [83–86]. Building on this work, we developed a whole-host model of Mtb infection called *HostSim*. *HostSim* is a multi-scale hybrid computational model that captures key features of pulmonary Mtb infection progression by representing the lungs, blood, and also an activated lymph node compartment [39]. *HostSim* adapted the architecture of our earlier LN and blood models and coupled it to a model of multiple lung granuloma formation [39,40]. Here, we use *HostSim* both to generate predicted trajectories of lung-sourced APCs and as a starting point for ODE development.

## 2. Model overview

To build a model of multiple LNs, we represent each LN with a system of ordinary differential equations (ODEs) that represent unique populations of antigen-presenting cells (APCs), T cells (different types), macrophages (different states), and Mtb (different locations) that was updated previously in the *HostSim* model [39,40]. Each term in the ODE system represents an immune cellular mechanism – i.e., a behavior, interaction, or transition – and their activity is characterized by one or more parameters (see S1 Appendix full list of equations). Our individual LNs can either remain *uninfected* (i.e., no APCs draining to that LN), participate in antigen presentation (i.e., become *activated* by the presence of antigen presentation cells) and/or form LN granulomas (i.e., become *diseased* in Mtb infection) (Fig 2B). When representing hosts with Mtb-infected LNs, we simulate two of five virtual LNs with a virtual host as being diseased; however, our model can readily adapt to include a larger number of diseased LNs per-host. In our model, blood serves as both a source of naïve or memory T cells trafficking to/from LNs and as a reservoir that T cells must travel through before trafficking to the site of primary infection (in the lungs). While we do not explicitly represent lungs in this model, we use *HostSim* to generate a time-course prediction of APC count from the lungs of virtual patients with either LTBI or experiencing an active pulmonary infection (Fig 3A and 3B) [39,40], which we then use as the source of APCs for our new multiple-diseased-LN model.

Our virtual population has N = 1000 hosts, each with five LNs. Given the range of infection presentations experimentally observed within LN infections and heterogeneity between LNs within individual Mtb hosts in both humans and NHPs [13], we define a unique set of parameters for each LN within a virtual host by employing our parameter sampling technique calibrated with data (see below). Our complete list of parameter ranges is found in **Tables A, B, and C in** **S2 Appendix**. For activated-LN simulations, we assume that pulmonary infection begins at simulation day 1. As simulations progress, individual LNs participate in antigen presentation, independently from one another. For diseased-LN simulations, we further let two LNs per-host become *diseased* (i.e., harbor live bacteria and form LN granulomas). We simulate each of our virtual hosts for 481 days post-infection (~16 months) to capture dynamics of both early and late-stage infection.

## 3. Creating the multiple lymph node model

Within a single host, LNs vary widely in their individual baseline characteristics, such as proximity to the site of infection, efficiency contributing to adaptive immunity, and ability to manage the presence of live Mtb bacilli and control it. We represent each LN in our model by an individual set of non-linear ODEs (S1 Appendix) and these ODEs are independent from each other via parametrization (Tables A, B, and C in S2 Appendix). We capture the dynamics of five lymph nodes within each host. We make this assumption as there is an average of 4–21 LN, with the average being 12 within a thoracic cavity of *Cynomolgus macaques* [13] and, on average, 5 thoracic lymph nodes are detectable during Mtb infection [8,13]. Diseased LNs (with potential to form LN granulomas) have three main outcomes: [1] sterilization, with Mtb bacteria clearing and no presence of granuloma formation, [2] controlled granuloma formation occurs, leading, stable levels of bacterial burden, and [3] uncontrolled infection, wherein the granuloma completely effaces LN structure (**Fig 1A**).

To summarize mechanisms that we define in our model equations (see Fig 3C-E), we assume that each virtual host is at healthy equilibrium prior to infection. We represent this state by five sets of ODEs, each representing one of the virtual host’s lung-draining LNs. Each of a host’s virtual LN connects to a single, well-mixed blood compartment. The blood compartment serves as a sole source of circulating T cells for LNs. Both Mtb-specific and Mtb-non-specific cells efflux from a LN into blood and from blood to LNs (initially we begin with only Mtb non-specific T cells) (see Fig 2C). Within each LN, there are populations of naïve and central memory CD4 + and CD8 + Mtb-specific (once APCs begin to arrive) and Mtb non-specific T cells (see Fig 2D). A complete list of abbreviations used for each cell type can be found in Table 2. We additionally describe full model equations and details for each in S1 Appendix.

## 4. Antigen-presenting cells

In response to ongoing lung infection, APCs traffic into all five LNs. We determine APC trafficking dynamics by two vectors: one representing typical dynamics of antigen presentation for LTBI hosts and another representing typical dynamics of antigen presentation during hosts with active pulmonary Mtb infection (Fig 3A and 3B). We derive the LTBI vector by averaging the number of APCs generated by 25 virtual LTBI hosts from our whole-host level model [39]. We derive the active Mtb infection vector from the HostSim model simulations by selecting a representative virtual host with active Mtb infection (in this case the host had 2 granulomas with very high bacterial loads even though others cleared or controlled, S1 Fig) [from 20]. The number of APCs for either LTBI or active APC vector is divided evenly among our 5 individual LNs to represent trafficking to individual LNs. While each LN receives the same number of APCs, LN responses differ due to small, biologically relevant variation in parameter values describing intra-LN behaviors (i.e., priming rates, T-cell proliferation rates). Once APCs arrive at a LN, naïve T cells are primed and differentiate into effector, effector memory, and central memory T cells. In response to APC encounters, memory T cells differentiate into effector T cells. Following these processes, T efflux (leave) from LNs and transit through blood to lungs, the site of original infection (see Fig 3D). Here, we track the number of cells leaving over time, but since we do not model the lung, we collect the cells over time in the vector for analysis representing functionality of LNs.

## 5. Model of a granuloma developing within a lymph node

To represent the ability of granuloma formation within LNs, we created a sub-model (granuloma compartment within a LN) representing key cell types found within LN granulomas. Specifically, we represent three types of macrophages (resting, infected, and activated), two types of Mtb (intracellular and extracellular), and two subsets of effector T cells (CD4 + and CD8+), hereafter referred to as *granuloma-associated T cells* (Fig 2D). A complete list of cell types within our model LN granulomas and their associated abbreviations can be found in Table 2.

We initiate LN granuloma formation with a single infected macrophage containing a single live intracellular Mtb. In our simulations, we initiate LN granulomas in two of five LNs 20 days after lung infection. We base this number on data derived from previous NHP experimental studies showing that in a given NHP host, 20–50% of LNs will be CFU+ [13], Therefore 2 of 5 modeled LNs is consistent with this observation. Previous studies support this timing and show that starting at approximately 21 days post-infection, viable bacteria are detectable within LNs [13].

Following this introduction, granulomas begin to form (or not) through interplay of macrophage, bacterial, and T-cell subtypes as described by the following equations:


$$\frac{d}{dt}M_{R}=\underset{\text{Recruitment}}{\underbrace{\alpha_{4a}\left(M_{A}+w_{2}M_{I}\right)\left(1-\frac{M_{R}}{n_{2}}\right)}}-\underset{\text{Macrophage Infection}}{\underbrace{k_{2}M_{R}\left(\frac{B_{E}}{B_{E}+c_{9}}\right)}}-\underset{\text{Macrophage activation}}{\underbrace{k_{3}M_{R}\left(\frac{B_{E}+w_{1}B_{I}}{B_{E}+w_{1}B_{I}+c_{8}}\right)\left(\frac{G_{4}}{G_{4}+hs_{4}}\right)}}-\underset{\text{Natural death}}{\underbrace{\mu_{M_{R}}M_{R}}}$$

$$\frac{d}{dt}M_{I}=\underset{\text{Macrophage infection}}{\underbrace{k_{2}M_{R}\frac{B_{E}}{B_{E}+c_{9}}}}-\underset{\text{Macrophage bursting}}{\underbrace{k_{17}M_{I}\left(\frac{B_{I}^{2}}{B_{I}^{2}+{\left(n_{1}M_{I}\right)}^{2}}\right)}}-\underset{\text{T-cell driven apoptosis}}{\underbrace{k_{52}M_{I}\left(\frac{G_{8}\left(\frac{G_{4}}{G_{4}+c_{E_{4}}}\right)+w_{1}G_{4}}{G_{8}\left(\frac{G_{4}}{G_{4}+c_{E_{4}}}\right)+w_{1}G_{4}+M_{I}c_{52}}\right)}}-\underset{\text{Natural death}}{\underbrace{\mu_{M_{I}}M_{I}}}$$

$$\frac{d}{dt}M_{A}=\underset{\text{Macrophage activation}}{\underbrace{k_{3}M_{R}\left(\frac{B_{E}+w_{1}B_{I}}{B_{E}+w_{1}B_{I}+c_{8}}\right)\left(\frac{G_{4}}{G_{4}+hs_{4}}\right)}}-\underset{\text{Natural death}}{\underbrace{\mu_{M_{A}}M_{A}}}$$

$$\begin{array}{cc} \frac{d}{dt}B_{I} & =\underset{\text{Intracellular replication}}{\underbrace{\alpha_{19}B_{I}\left(1-\frac{\frac{B_{I}}{M_{I}}}{N_{1}}\right)}}+\underset{\text{Macrophage infection}}{\underbrace{k_{2}\frac{N_{1}}{2}M_{R}\left(\frac{B_{E}}{B_{E}+c_{9}}\right)}}-\underset{\text{Macrophage bursting}}{\underbrace{k_{17}N_{1}M_{I}\left(\frac{B_{I}^{2}}{B_{I}^{2}+{\left(n_{1}M_{I}\right)}^{2}}\right)}}\\ & \;-\underset{\text{T-cell driven apoptosis of }M_{I}}{\underbrace{k_{52}M_{I}\frac{B_{I}}{M_{I}}\left(\frac{G_{8}\left(\frac{G_{4}}{G_{4}+c_{E_{4}}}\right)+w_{1}G_{4}}{G_{8}\left(\frac{G_{4}}{G_{4}+c_{E_{4}}}\right)+w_{1}G_{4}+M_{I}c_{52}}\right)}}-\underset{\text{Natural death}}{\underbrace{\mu_{B_{I}}B_{I}}}-\underset{\text{Release of }B_{I}\text{ by naturally dying }M_{I}}{\underbrace{\mu_{M_{I}}\frac{B_{I}}{M_{I}}M_{I}}} \end{array}$$

$$\begin{array}{cc} \frac{d}{dt}B_{E} & =\underset{\text{Extracellular replication}}{\underbrace{\alpha_{20}B_{E}\left(1-\frac{B_{E}}{N_{3}}\right)}}+\underset{\text{Release of }B_{I}\text{ by naturally dying }M_{I}}{\underbrace{\mu_{M_{I}}\lambda_{surv}B_{I}}}+\underset{\text{Macrophage bursting}}{\underbrace{k_{17}N_{I}M_{I}\left(\frac{B_{I}^{2}}{B_{I}^{2}}+{\left(n_{1}M_{I}\right)}^{2}\right)}}\\ & \;+\underset{\text{T-cell driven apoptosis of }M_{I}}{\underbrace{k_{52}N_{fracc}B_{I}\left(\frac{G_{8}\left(\frac{G_{4}}{G_{4}+cE_{4}}\right)+w_{1}G_{4}}{G_{8}\left(\frac{G_{4}}{G_{4}+c_{E_{4}}}\right)+w_{1}G_{4}+M_{I}c_{52}}\right)}}-\underset{\text{Macrophage infection}}{\underbrace{k_{2}\frac{N_{1}}{2}M_{R}\left(\frac{B_{E}}{B_{E}+c_{9}}\right)}}\\ & \;-\underset{\text{Activated macrophage killing of }B_{E}}{\underbrace{k_{15}M_{A}B_{E}}}-\underset{M_{R}\text{ killing of }B_{E}}{\underbrace{k_{18}M_{R}B_{E}}}-\underset{\text{Natural death}}{\underbrace{\mu_{B_{E}}B_{E}}} \end{array}$$

$$\frac{d}{dt}G_{4}=\underset{\text{Recruitment from LN}}{\underbrace{\xi_{3}E_{4}\frac{w_{2}M_{I}+M_{A}}{w_{2}M_{I}+M_{A}+hs_{6}}}}+\underset{\text{Proliferation}}{\underbrace{k_{9}G_{4}\left(\frac{\rho_{2}}{G_{4}+\rho_{2}}\right)\left(\frac{M_{I}}{M_{I}+hs_{6}}\right)}}$$

$$\frac{d}{dt}G_{8}=\underset{\text{Recruitment from LN}}{\underbrace{\xi_{9}E_{8}\frac{w_{2}M_{I}+M_{A}}{w_{2}M_{I}+M_{A}+hs_{8}}}}+\underset{\text{Proliferation}}{\underbrace{k_{19}G_{8}\left(\frac{\rho_{3}}{G_{8}+\rho_{3}}\right)\left(\frac{M_{I}}{M_{I}+hs_{6}}\right)}}.$$

Here, we show an example of the equations describing LN granuloma formation for one LN with definitions of individual model states given in Table 2. The full set of model equations describing all LNs can be found in S1 Appendix and a complete description of model parameters can be found in Tables A, B, and C in S2 Appendix.

Within a virtual LN, resting macrophages are recruited to a forming granuloma via signals from existing infected and activated macrophages in the LN granuloma. Resting macrophages are either infected through Mtb uptake or activated by CD4 + T cells. If infected, macrophages serve as a replicative niche for Mtb and either burst due to intracellular bacterial overload or undergo apoptosis following T-cell signaling. Alternatively, if activated, macrophages participate in extracellular bacterial killing. Mtb exists in one of two states: intracellular (within) or extracellular (outside of) infected macrophages. When intracellular, Mtb replicate and are released into the environment following macrophage bursting or natural death. They are also subject to natural death or macrophage-mediated killing. When extracellular, Mtb replicate and are subject to uptake by macrophages. Extracellular Mtb can also undergo activated- and resting-macrophage-mediated killing as well as, in rare cases, natural death. Granuloma-associated CD4 + and CD8 + T cells are recruited to a developing granuloma and proliferate based on infected and activated macrophage cell counts that represent a proxy for cytokine signaling produced by each cell, respectively. While there are no definitive data that T cell proliferation occurs within LN granulomas, a secondary source of T cells is necessary in our model formulation to capture experimentally-measured T-cell counts. Once diseased within a LN granuloma, granuloma-associated T cells are unable to leave the LN.

## 6. Virtual host death

Within our model, we do not explicitly model physiological attributes such as strength of LN walls. This means that our virtual LNs can reach cellular levels and infection severity that is not clinically relevant, and these virtual LNs would result in LN bursting and animal death if they were within an NHP, for example. To account for this, we assume that our virtual hosts die at the first time point that a virtual LN exceeds 10^7^ CFU [9]. We do not plot outcomes after day of virtual death under the assumption that any data thereafter is not clinically relevant. While this is reasonable for all clinically relevant analyses, we chose to these values in sensitivity analyses because they allow us to see extremes of disease progression and drivers of the underlying dynamics resulting in death.

## 7. Calibration data

Most calibration data comes from a single study published by the Flynn lab [13]. In this study, 32 Cynomolgus macaques were infected with a low dose (~1–28 CFU) of Mtb strain Erdman. At necropsy, LNs were excised and cut into two sections. One section was homogenized into a single cell suspension for immunological testing and aliquots made to obtain colony forming units (CFU). The other section was prepared for histologic examination. For immunological testing, single cell suspensions were stimulated with Mtb specific antigens ESAT-6 and CFP-10 in presence of Brefeldin A and, separately, were stimulated with non-specific antigens phorbol dibutyrate (PDBu) and ionomycin. The flow cytometry panel for these samples examined cell surface markers CD3, CD4, and CD8 and intracellular staining for cytokines IL-2, TNF, IFNg, IL-17, and IL-10. Histological examination was performed by an experienced veterinary pathologist with characteristics of granulomas being noted. See [13] for complete details on data collection methods.

Aside from this study, we calibrate our model activated (no antigen presentation or LN granuloma formation) LNs to known healthy T cell concentrations within blood of *Cynomolgus macaques* [87] and an estimated number of total T cells within individual LNs (see subsection below). Additionally, total model CD4 + T cells and total CD8 + T cells in blood are calibrated to cellular blood concentrations from [46].

From the Flynn lab study [13], we have access to data at the resolution of individual NHP LNs and the number of cells within them (which were presented as avg in the original study). We assume that all NHP LNs we have data for are activated (receiving APCs) and/or diseased (containing a LN granuloma) because, if non-activated (not receiving APCs), they are not enlarged enough to be chosen for excision. In this dataset, there are some NHPs that have multiple LNs with complete data. We treat each LN as independent regardless of origin because it is known that LNs have different responses to Mtb infection even within the same host. We classify each NHP LN as activated (receiving APCs) if a LN was both colony-forming unit (CFU) negative and lacked a granuloma on gross pathology inspection. If these conditions are not met, we classify a LN as diseased (receiving APCs and containing a LN granuloma). For calibration, we map data from each of these classifications of NHP LNs to model LNs of the same name and type.

Within each LN classifications, we calibrate NHP and model LN cell counts by comparing 6 unique datasets: total CD4 + T-cells, total CD8 + T-cells, Mtb-specific CD4 + T-cells, Mtb-specific CD8 + T-cells, total macrophages, and total Mtb. We assume cell count data from NHP LNs following stimulation with phorbol dibutyrate (PDBu) and ionomycin maps onto our virtual total (Mtb-specific and Mtb-nonspecific) LN CD4 + and CD8 + T cells, respectively. Additionally, we assume cell count data from NHP LNs following stimulation with ESAT-6 and CFP-10, Mtb-specific antigens, maps onto our virtual Mtb-specific LN CD4 + and CD8 + T cells, respectively. Total NHP LN macrophages map to total virtual LN macrophages and total NHP LN CFU map to total virtual LN Mtb.

Each LN within the dataset was also classified by a pathologist into two categories based on effacement status: greater than (>) 50% effacement and less than (<) 50% effacement. Greater than 50% effacement implies approximately greater than half of a LN is comprised of structures that were granulomatous material. Those that were less than 50% effacement meant that less than half (or none) of a LN contains granulomatous material. In our study, we use this classification to validate our model outcomes.

### 7.1. Immunohistochemistry

LNs from Mtb-infected thoracic LNs were stained as previously described in [88]. Briefly, thoracic LNs were harvested from animals being necropsied as part of ongoing studies and were fixed in 10% neutral-buffered formalin before being embedded in paraffin and sectioned at 5 mm/section. Sections were deparaffinized and antigen retrieval was performed as previously noted [88] and adjacent sections were stained for CD3 + T cells (rabbit polyclonal; Dako, Carpinteria, CA), and CD11c (mouse monoclonal, clone 5D11; Leica Microsystems, Buffalo Grove, IL), followed by fluorochrome-conjugated secondary antibodies. CD20 (rabbit polyclonal; Thermo Fisher Scientific, Waltham, MA) was stained with Invitrogen’s Zenon labeling kit (Thermo Fisher Scientific) as a directly conjugated tertiary. Adjacent sections were visualized for high endothelial venules (HEV) and lymphatic vessels, by staining for PNAd (clone MECA-79; BioLegend, San Diego, CA) and LYVE-1 (goat polyclonal; Biotechne, Minneapolis, MN) as well as CD3 T cells (Dako). The sections were imaged with either an Olympus Fluoview 500 or Fluoview 1000 laser scanning confocal microscope (Olympus, Center Valley, PA) maintained by the University of Pittsburgh’s Center for Biologic Imaging (Fig 1A) or a Nikon e1000 epifluorescence microscope (Nikon Instruments, Melville, NY) (Fig 1B). Three-color images (red, green, far red [pseudocolored as blue]) were acquired sequentially at 20x magnification, followed by a DAPI image (gray) showing nuclei. Because the lymph nodes were too large to image in a single field, multiple overlapping fields were acquired and assembled into a single composite image with Photoshop (Adobe Systems Incorporated, San Jose, CA) or Nikon Elements AR.

### 7.2. Estimation of LN T cell counts in healthy NHPs

For validation of our model in the absence of Mtb infection, we estimate the number of CD4 + and CD8 + T cells within a LN. Experimentally, it is difficult to detect non-stimulated LNs and verify whether they all contain similar numbers of T cells. This effect is further confounded by LN size variability upon antigen presentation. To create an estimate, we list a number of assumptions and published data from literature below.

1. We assume that naïve T-cell repertoires described below is scalable by weight between *Cynomolgus macaques* and humans to estimated T-cell counts in uninfected LNs. NHP weight is approximately a tenth of a human’s body weight [89]. This gives a human naïve T-cell repertoire (approximately 3*10^11^ total naïve T cells across both CD4 + and CD8+) [90], and we infer an average NHP naïve T-cell repertoire size of 3*10^10^ across both CD4 + and CD8 + T cells. Similar comparisons have been made between mice and humans [90].
2. We consider 60% of naïve T cells to be CD4 + and 40% to be CD8+ [90].
3. We assume that a majority (50–100%) of the total LN T-cell population is naïve (not specific to any particular antigen measured here) [90].
4. We assume 49% of naïve T-cell populations are within LNs at any given time for the following two reasons: First, we assume that lymphatic tissues contain populations of T cells within the spleen, lymph nodes, and tertiary lymph nodes. We assume that half of T cells within lymphatic tissues reside within the spleen because approximately half of T cells secreted into blood come from the spleen; we also assume that a negligible portion of the T-cell population resides within tertiary lymphoid structures. This means that approximately 50% of T cells within lymphatic tissues lie in LNs. Moreover, at any given time, 98% of the CD4 + and CD8 + T-cells are circulating through lymphatic tissue [91]. (rather than blood). We obtain 49% as the product of these estimations.
5. We assume that lymph influx may be as low as 10% of the lymphatic system’s capacity [92].
6. In absence of published NHP counts, we assume that numbers of LNs within NHPs are between 100 and 800 (fewer than or comparable to human LN counts) and assume that LDLNs have near-average T-cell population sizes.
7. We assume between 1:200,000 and 1:2,000,000 (CD4+) and 1:20,000 and 1:1,300,000 (CD8+) T cells will respond to Mtb antigen [90] (i.e., will be Mtb-specific in our model).
8. Datasets will include additional variation on the order of > 30%, due to environmental or behavioral factors [93]. We capture this below as increasing or decreasing the above estimates by 15%.

Factoring these together, we calculate the following estimates for T-cell counts within individual LNs:


$$\text{Mtb-specific CD4+ Upper Bound}=\left(3*10^{10}\right)*60\;\%*(50\;\%{)}^{-1}*49\;\%*100\;\%*\frac{1}{100}*1:\left(2*10^{-5}\right)*1.15\approx 4000$$

$$\text{Mtb-specific CD4+ Lower Bound }=\left(3*10^{10}\right)*60\;\%*(100\;\%{)}^{-1}*49\;\%*10\;\%*\frac{1}{800}*1:\left(2*10^{-6}\right)*0.85\approx 2$$

$$\text{Mtb-specific CD8+ Upper Bound}=\left(3*10^{10}\right)*40\;\%*(50\;\%{)}^{-1}*49\;\%*100\;\%*\frac{1}{100}*1:\left(2*10^{-4}\right)*1.15\approx 27000$$

$$\text{Mtb-specific CD8+ Lower Bound }=\left(3*10^{10}\right)*40\;\%*(100\;\%{)}^{-1}*49\;\%*10\;\%*\frac{1}{800}*1:\left(1.3*10^{-6}\right)*0.85\approx 1$$

$$\text{Nonspecific CD4+ Upper Bound }=\left(3*10^{10}\right)*60\;\%*(50\;\%{)}^{-1}*49\;\%*\frac{1}{100}*1.15\approx 10^{8}$$

$$\text{Nonspecific CD4+ Lower Bound }=\left(3*10^{10}\right)*60\;\%*(100\;\%{)}^{-1}*49\;\%*10\;\%*\frac{1}{800}*0.85\;\approx 10^{6}$$

$$\text{Nonspecific CD8+ Upper Bound }=\left(3*10^{10}\right)*40\;\%*(50\;\%{)}^{-1}*49\;\%\;*\;100\;\%*\frac{1}{100}*1.15\approx 10^{8}$$

$$\text{Nonspecific CD8+ Lower Bound }=\left(3*10^{10}\right)*60\;\%*(100\;\%{)}^{-1}*49\;\%*10\;\%*\frac{1}{800}*0.85\approx 6*10^{5}$$

## 8. Parameter estimation and model calibration.

The goal of our calibration is to isolate a large range of parameters that generate heterogeneous model outcomes that are not falsifiable through any available data. In this way, we seek to capture a range of outcomes wider than those seen *in vivo* while remaining consistent with experimental observations. As we used *HostSim* LN and blood ODEs as a starting point for the individual LN ODEs and used *HostSim* lung granuloma ODEs as a starting point for the LN granuloma ODEs, we began simulations by using parameter ranges in those original model equations for our updated individual LN granuloma model [39]. We employed two primary methodologies to modify our published, previous parameter ranges, and we describe both in brief below.

### 8.1. Calibration protocol using Latin hypercube sampling

The goal of calibration is to tune model parameters so that model outputs recapitulate variation observed in target datasets [94,95]. We performed calibration using our CaliPro method [90] and summarize our application of it here. 500 combinations of model parameters are globally sampled from uniform distributions using a technique called Latin hypercube sampling (LHS) [91]. Using these samples, parameters are grouped into either “pass” or “fail” sets depending on whether model outputs match target datasets as follows. Consistent with published CaliPro examples [89,90] at each timepoint in our datasets we widen a dataset range by a magnitude to specify a pass set definition; this prevents simulations that do not strictly match a dataset range from being excluded to allow for subsequent improvement. When the pass rate of sampled parameters exceeds 90%, calibration process is stopped to not overfit the data [90]. To improve pass rate between calibration iterations, parameter ranges under calibration are adjusted using a technique of alternative density subtraction, which subtracts a fail parameter set probability density from a pass parameter set probability density [90]. Note that we do not fix parameter values even when performing model calibration to capture biological variability between LNs, hosts, and granulomas. In total, 74 parameters across five LNs are varied, and 22 parameters are fixed.

### 8.2. CaliPro, the Calibration protocol, uses visual inspection and identification

The above-described calibration protocol, CaliPro [90], is unable to account for pass sets not capturing within data ranges. Thus, we augment the calibration protocol approach by employing a method that uses visually identifiable hosts with favorable characteristics. Specifically, “good hosts” are those whose outcomes are closer to the median of calibration data. We then determine, for each of these “good hosts”, where in a previous parameter range a host parameters fell. If any of those “good parameter values” fell near an edge of their source parameter range (within 10% of an edge of the range), we expanded and recentered the parameter’s range to center around that “good value” in the logarithmic scale. We continue this process iteratively until sampling ranges produce model results that adequately capture data ranges.

### 8.3. Model scope

The scope of a model is the set of all credible statements that a model can make and highly is related to the set of mechanisms validated within the model. Systematic assessment of a model’s full scope is beyond the purview of this paper. Rather, we determine whether claims about individual outcomes are within-scope by determining (i) if known biologically-relevant mechanisms have been explicitly represented while justifying simplifications, and (ii) if a model can reproduce datasets and qualitative behaviors that were not used for calibrate (i.e., model validation). As Mtb infection is chronic and potentially lasting for decades, we assume that trajectories that exhibit slow long-term changes (other than sterilization) are reasonable, and therefore predictions beyond 200 dpi to be within-scope. Within the results section we explicitly indicate results we are using as validation.

Note that, while we simulate multiple biologically-relevant spatial scales (i.e., cell, tissue, and host), we do not explicitly represent spatial gradients of molecules within any individual model component. This is because we find a non-spatial model to be both feasible and sufficient for our goal: to simulate longitudinal trajectories of LN granuloma infection and determine biological mechanism influential over LN and LN granuloma outcomes.

## 9. Outcome measures

To best determine mechanisms that may predict LN granuloma fates we define the following output measures:

### 9.1. LN granuloma bacterial load

For each individual, diseased (LN granuloma-containing) LN, we sum bacteria counts over all subtypes. *This determines LN granuloma fate.*

For some of our analyses, we assign each simulation as having one of three fates: bacterial levels that are growing large, bacterial loads that are stable, and bacterial levels that sterilize. We define bacterial levels that are growing large as those that have a maximum bacterial load at the end of simulation period. We define bacterial loads that are stable as those that a bacterial load remains greater than 0.5 and have reached a maximum bacterial load before the end of the simulation. Lastly, we define LN granulomas that sterilize as those that have a bacterial load of less than 0.5 at any point during the simulation.

### 9.2. Time-to-sterilization

For all LNs undergoing granuloma formation, we define time-to-sterilization as the first time point after initial seeding of live bacteria within LNs that total bacterial load (regardless of intracellular status) fell below less than 0.5 bacteria. Note that any rebounds in bacterial loads above 0.5 we disregard as an artifact of using a continuous model.

We also use time-to-sterilization to capture how non-sterilizing LN granulomas will take longer to clear than the study duration, if at all. To this end, we default time-to-sterilization to day 482 for all non-sterilizing granuloma-forming LNs, although any arbitrary time beyond the simulation end-time yields the same PRCC results. This is because PRCC uses Spearman correlations, and consequently all non-sterilizers are ranked identically.

### 9.3. Serial 2-deoxy-2-[18F]-D-deoxyglucose (FDG) avidity

PET/CT scans are a non-invasive method of examining granulomas. Scans using 18F-fluorodeosyglucose are used to measure metabolic activity of a tissue [44,45]. We do not explicitly model metabolic activity within our LN; however, we approximate FDG avidity as a weighted sum of cell counts, where more metabolically active cell types are more highly weighted. Simulated FDG avidity is an exploratory measurement of metabolic activity adapted from our previous work [4]. This measurement assumes that pro-inflammatory cell states are more metabolically active and resting/memory/non-effector states are less metabolically active. Factors that influence real FDG avidity are currently not experimental known and thus we hypothesize that relative cellular activity based on numbers are a fair proxy.

We made four assumptions: (i) activated macrophages are more metabolically active than infected macrophages; (ii) activated macrophages were 1.5x more metabolically active than effector T cells; (iii) that metabolic activity level of T cells was greatest in effector cells, less in memory cells, and further less in precursor cells; and (iv) that CD4 + and CD8 + T cells had similar levels of metabolic activity. Our weights, given below, reflect these assumptions. Note that scaling the entire measurement up or down does not affect our conclusions because our analysis, PRCC, is a method that ranks outcomes relative to one another (rather than using the absolute levels of sFDG). The calculation is as follows and can be modified as new data are available.

$sFDG\;=\;2P_{4}+4E_{4}+4G_{4}+3CM_{4}+3EM_{4}+2P_{8}+4E_{8}+4G_{8}+3CM_{8}+3EM_{8}+5M_{I}+6M_{A}$

### 9.4. Numbers of effluxing T-cells

For all LNs, we define numbers of effluxing T cells as the number of Mtb-specific effector T-cells (CD4 + and CD8+) that leave a given LN at a time point.

### 9.5. Virtual lymph node effacement

In all diseased LNs that undergo granuloma formation, we calculate percent effacement and bin it into two categories: greater than (>) 50% effacement and less than (<) 50% effacement. To find percent effacement, we take volume of total granuloma-associated cells (macrophages, granuloma-associated T cells, and Mtb) and divide it by total LN volume (i.e., granuloma-associated cells and non-granuloma-associated LN T-cells). We assumed that macrophages, T cells, and LNs are approximately spherical in shape and bacteria are approximately cylindrical. The specific formula used to calculate percent effacement is as follows:


$${\text{p}}_{\text{LN,eff}}=\frac{\underset{\text{LN granuloma volume}}{\underbrace{\left({\frac{4}{3}\pi \frac{{\text{d}}_{\text{M}}}{2}}^{3}{\text{M}}_{\text{tot}}\right)+\left({\frac{4}{3}\pi \frac{{\text{d}}_{\text{T}}}{2}}^{3}{\text{T}}_{\text{G,tot}}\right)+\left({l_{B}\pi \frac{d_{B}}{2}}^{3}B_{tot}\right)}}}{\underset{\text{Total LN volume}}{\underbrace{\left({\frac{4}{3}\pi \frac{{\text{d}}_{\text{M}}}{2}}^{3}{\text{M}}_{\text{tot}}\right)+\left({\frac{4}{3}\pi \frac{{\text{d}}_{\text{T}}}{2}}^{3}{\text{T}}_{\text{G,tot}}\right)+\left({{\text{l}}_{\text{B}}\pi \frac{{\text{d}}_{\text{B}}}{2}}^{3}{\text{B}}_{\text{tot}}\right)+\left({\frac{4}{3}\pi \frac{{\text{d}}_{\text{T}}}{2}}^{3}{\text{T}}_{\text{NG,tot}}\right)}}}$$

Where $M_{tot}$ is total number of macrophages within a LN granuloma, $T_{G,tot}$ is number of granuloma-associated T cells, $B_{tot}$ is total number of bacteria within a LN granuloma, and $T_{NG,tot}$ is number of non-granuloma-associated T-cells. $d_{M}$, $d_{T}$, and $d_{B}$ correspond to the diameters of macrophages, T cells and Mtb, respectively and $l_{B}$ corresponds to length of Mtb.

We define LNs with a greater than 50% effacement to be those that have a percent effacement greater than or equal to 0.5 and LNs with less than 50% effacement to be those that have a percent effacement of less than 0.5. Given that almost all LNs in the NHP experimental dataset are from 201 days post-infection or shorter and that we assume that the majority of highly effaced LNs at late time point belong to NHPs that would have to be euthanized due to severe disease progression, we calculate virtual LN effacement values at 201 days post-infection.

## 10. Uncertainty and sensitivity analyses

To determine mechanisms driving key outcomes of interest as described above, we perform 2 quantitative statistical techniques called uncertainty and sensitivity analyses. Using Latin hypercube sampling, we efficiently sample our parameter ranges to generate 1000 virtual hosts. Given our individual LNs are independent copies of one another, we pool our LNs as either diseased or activated. This means that, for a diseased host, we have 2000 diseased virtual LNs and 3000 activated virtual LNs in our final analysis set. Then, to determine relative impact of changes to parameter values on model output measures of interest, we calculate correlations using the Partial Rank Correlation Coefficient (PRCC) method, a well-established method of determining correlation-based sensitivity [91].

In brief, PRCC is a method of assessing nonlinear correlations between model inputs (parameters) and a specific model output measure. As an example, a PRCC value indicates dependence of a variation of an outcome measure (e.g., total bacterial burden at a given timestep) on each parameter in a model. Because our model generates outcomes that we can measure at each time point, we use PRCC to assess correlations in both time and across parameters. We also perform Bonferroni corrections for multiple comparisons, given that we are determining the dependence of an outcome on each parameter simultaneously [89,91]. We do not expect that a single mechanism will have a large correlation, as this would be a biological fail-point. Moreover, PRCC values are partial-correlations, which remove the linear contribution and may mean that absolute correlation values appear smaller while still retaining biological significance [96].

To further simplify interpretation of our sensitivity analysis, post-PRCC analysis we calculate average PRCC value for each parameter in 50-day ranges. This is done to represent and visualize results and trends of data more easily (see **Results** for details). We also exclude from our analyses any parameters that have PRCC values that is significant for less than 30 days within a period. We do this because we assume that, if the PRCC value of a parameter is not significant for at least 30 days within a 50-day range, it is likely an artifact rather than a true result.

## 11. Model simulation and analysis tools

We implement our model code and preliminary data analysis in MATLAB (2024a). We solve our system of ODEs using MATLAB’s ode15s solver. Post-processing statistical analysis was performed within MATLAB (R2024a) and all figures were generated using R (R version 4.3.2). We also provide (i) an SMBL-encoded version of the ODE component of our model (generated using MOCCASIN [97]), (ii) spreadsheets containing parameter and initial condition ranges that we used (i.e., a machine-readable version of Tables A, B, and C in S2 Appendix); and (iii) the specific per-virtual-host parameter and initial conditions we used for all simulations presented in this work. Hyperlink: http://malthus.micro.med.umich.edu/lab/lymphSim/

## Supporting information

S1 Appendix Model ODE Equations. This document contains the equations used in our multi-LN model of the LDLN response to pulmonary Mtb infection. These equations are split up into three classes of systems: blood 7 equations (Section 1), lymph node equations (Section 2), and LN granuloma equations (Section 3).
(PDF)

S2 Appendix Model Parameters. This appendix provides a complete list of model parameters for equations given in S1 Appendix. **Table A** details blood parameters. **Table B** details lymph node parameters. **Table C** details lymph node granuloma parameters. Column 1 shows the 5 searchable name of each parameter. Bl refers to blood. Column 2 shows the symbol used in the equations. Column 3 givens a 6 description of the parameter. The last 3 columns refer to the uncertainty analysis parameter distributions and the range values of minimum and maximum.
(PDF)

S1 Text Supplementary Model Information. This document details pulmonary status of actively infected host (**Fig A**), model blood (**Fig B**) and negative control calibration (**Fig C**), and actively infected host analyses that parallel the LTBI host presented in the manuscript (**S1-**S5 Figs).
(PDF)

S1 Fig Lung granuloma output for active pulmonary disease host. Model output for granulomas from a representative host that was used to generate APCs from a host with active pulmonary disease. Shown are cell numbers and bacterial levels for this representative active host (colors represent unique granuloma trajectories within our representative host). Two granulomas (above in purple) have high-burden, uncontrolled bacteria indicating active granulomas, and thus an active pulmonary infection. All other granulomas (other lines) are granulomas where bacteria are controlled or cleared.
(TIF)

S2 Fig Multiple-LN model captures expected evolution of immune T cell population dynamics from the blood compartment in 3 cases: uninfected, activated, and diseased lymph nodes. We simulated 1000 virtual hosts having both LTBI and active pulmonary disease (using the unique APC trajectories respectively). Our model is calibrated to capture key dynamics of total T cells in the blood within the uninfected (A, D), activated (B, E), and diseased (C, F) cases for virtual hosts with LTBI (A, B, C) and virtual hosts with active pulmonary infection (D, E, F). Uninfected hosts have no Mtb infection and no APC driven activation in their LNs. Activated hosts have five LNs receiving Mtb activated APCs. Diseased hosts have five activated LNs receiving Mtb activated APCs and LN granulomas forming in LN #1 and #2. We simulate 1000 separate virtual hosts for each case. Black dashed line in A and B represents average concentration of CD4 + and CD8 + T-cells in blood of a healthy animal. Flow cytometry data from individual NHPs is represented by black dots from [13] in B, C, E, and F.
(TIF)

S3 Fig Multiple-LN model captures expected evolution of immune T cell population dynamics in uninfected lymph nodes for 1000 virtual hosts. Our model is calibrated to capture key dynamics of Mtb-specific T cells (A, C) and total T cells (B, D) for virtual uninfected hosts with both LTBI (A, B) and active pulmonary infection (C, D). Uninfected hosts have no Mtb infection and no APC-driven activation in their LNs. We simulated 1000 separate virtual hosts for each case. In each plot, 1000 hosts are represented, each host LN is a line.
(TIF)

S4 Fig Multiple-LN model captures expected evolution of immune T cell population dynamics in activated and diseased cases for 1000 virtual hosts with active pulmonary disease. Our model is calibrated to capture key dynamics of Mtb-specific T cells (A, C) and total T cells (B, D) within activated (A, B) and diseased (C, D) cases. Activated hosts have five LNs receiving Mtb activated APCs. Diseased hosts have five activated LNs receiving Mtb activated APCs and LN granulomas forming in LN #1 and #2. For diseased LNs, our model captures the dynamics of LN bacterial load (E) and macrophages (F). We simulated 1000 separate virtual hosts for each case, generating a distinct trajectory for each of their LNs based on their parameterization. Lines in each plot show cell populations from the indicated LN within one host. For LN bacterial load (E) and macrophages (F), lines are colored by bacterial load trajectory: growing large (purple lines), stabilization (teal lines), and sterilization (yellow lines). Flow cytometry data from individual NHP LNs taken at necropsy are represented by black dots from [13]. Note that lines are truncated on virtual host death (see **Methods,** Section 6).
(TIF)

S5 Fig Bacterial load is driven by a balance of macrophage infection and activation within 1000 hosts with active pulmonary disease. (A) Proportion of 2000 virtual LN granulomas by fate: no bacteria present (sterilized), stable bacterial growth (stable), and uncontrolled bacterial growth at 481 days post lung infection (N = 2000). (B) Summary of sensitivity analysis detailing significant parameters driving total bacterial load. PRCCs are binned into 50-day bins for ease of analysis (see Methods). Shading indicates average PRCC value during a time interval *t* (given a parameter is at least significant for 30 days in *t*). A blue color indicates a positive correlation, and red color indicates a negative correlation. Significance alpha = 0.01 after Bonferroni correction. Complete model state descriptions (MR, MI, E4, etc.) can be found in Table 2 in Methods and parameter value description found in **Tables A, B, and C in** S2 Appendix.
(TIF)
