# Supplementary material for: Distinguishing multiple roles of T cell and macrophage involvement in determining lymph node fates during Mycobacterium tuberculosis infection
Source: PLoS Comput Biol. 2025 May 7;21(5):e1013033. doi: 10.1371/journal.pcbi.1013033 (PMC12084042; doi:10.1371/journal.pcbi.1013033)
Supplement: S1 Appendix — This document contains the equations used in our multi-LN model of the LDLN response to pulmonary Mtb infection. These equations are split up into three classes of systems: blood 7 equations (Section 1), lymph node equations (Section 2), and LN granuloma equations (Section 3). (PDF) [file pcbi.1013033.s001.pdf]

S1 Appendix for

Distinguishing multiple roles of T cells and macrophage involvement in determining lymph node fates during *Mycobacterium tuberculosis* infection

K.C. Krupinsky, C.T. Michael, P. Nanda, J. Mattila, D. Kirschner

This document contains the equations used in our multi-LN model of the LDLN response to pulmonary Mtb infection. These equations are split up into three classes of systems: blood equations (Section 1), lymph node equations (Section 2), and LN granuloma equations (Section 3). In this study, we used five lymph nodes which we differentiate with the index ℓ. For example, $\frac{dP_4^\ell}{dt} = \cdots$; is the equation for the CD4+ precursor T-cell population for lymph node ℓ.

Below we present, for convenience, a list of state variables for both blood and lymph node equations including a PDF-searchable reference (e.g. the concentration N_4^B has searchable term "BIN4C").

**Blood State Variables**<br>*All units in cells/ml*		
**State Variable**	**Searchable name**	**Description**
N_4^B	BIN4	Mtb-specific CD4+ Naïve T cell concentration
E_4^B	BIE4	Mtb-specific CD4+ Effector T cell concentration
CM_4^B	BICM4	Mtb-specific CD4+ Central Memory T cell concentration
EM_4^B	BIEM4	Mtb-specific CD4+ Effector Memory T cell concentration
N_8^B	BIN8	Mtb-specific CD8+ Naïve T cell concentration
E_8^B	BIE8	Mtb-specific CD8+ Effector T cell concentration
CM_8^B	BICM8	Mtb-specific CD8+ Central Memory T cell concentration
EM_8^B	BIEM8	Mtb-specific CD8+ Effector Memory T cell concentration
$N_4^{B,Non}$	BIN4Non	Non-specific CD4+ Naïve T cell concentration
$E_4^{B,Non}$	BIE4Non	Non-specific CD4+ Effector T cell concentration
$CM_4^{B,Non}$	BICM4Non	Non-specific CD4+ Central Memory T cell concentration

$EM_4^{B,Non}$	BIEM4Non	Non-specific CD4+ Effector Memory T cell concentration
$N_8^{B,Non}$	BIN8Non	Non-specific CD8+ Naïve T cell concentration
$E_8^{B,Non}$	BIE8Non	Non-specific CD8+ Effector T cell concentration
$CM_8^{B,Non}$	BICM8Non	Non-specific CD8+ Central Memory T cell concentration
$EM_8^{B,Non}$	BIEM8Non	Non-specific CD8+ Effector Memory T cell concentration

**Lymph Node State Variables** *All units in cell counts*		
**State Variable**	**Searchable name**	**Description**
APC^{ℓ}	APC	Antigen-presenting cells
N_4^{ℓ}	LnN4	Mtb-specific CD4+ Naïve T cells
P_4^{ℓ}	LnP4	Mtb-specific CD4+ Precursor T cells
E_4^{ℓ}	LnE4	Mtb-specific CD4+ Effector T cells
CM_4^{ℓ}	LnCM4	Mtb-specific CD4+ Central Memory T cells
EM_4^{ℓ}	LnEM4	Mtb-specific CD4+ Effector Memory T cells
N_8^{ℓ}	LnN8	Mtb-specific CD8+ Naïve T cells
P_8^{ℓ}	LnP8	Mtb-specific CD8+ Precursor T cells
E_8^{ℓ}	LnE8	Mtb-specific CD8+ Effector T cells
CM_8^{ℓ}	LnCM8	Mtb-specific CD8+ Central Memory T cells
EM_8^{ℓ}	LnEM8	Mtb-specific CD8+ Effector Memory T cells
$N_4^{\ell,Non}$	LnN4Non	Non-specific CD4+ Naïve T cells
$CM_4^{\ell,Non}$	LnCM4Non	Non-specific CD4+ Central Memory T cells
$N_8^{\ell,Non}$	LnN8Non	Non-specific CD8+ Naïve T cells
$CM_8^{\ell,Non}$	LnCM8Non	Non-specific CD8+ Central Memory T cells

**Lymph Node Granuloma State Variables** *All units in cell counts*		
**State Variable**	**Searchable name**	**Description**
M_R^{ℓ}	MR	Resting macrophage
M_I^{ℓ}	MI	Infected macrophage
M_A^{ℓ}	MA	Activated macrophage
B_I^{ℓ}	BI	Intracellular bacteria
B_E^{ℓ}	BE	Extracellular bacteria
G_4^{ℓ}	G4	CD4+ Granuloma-involved T cells
G_8^{ℓ}	G8	CD8+ Granuloma-involved T cells

1 Blood equations

BIN4C - N_4^B: The concentration of naive CD4+ T cells circulating in blood. These cells are produced by the thymus at a virtual-host dependent rate. They also efflux from / influx to each lymph node and are recruited to lymph nodes via cytokine signals. APCs serve as a proxy for the cytokine levels produced in the LNs. These cells also die in the blood at a per-host death rate μ_8

$$\frac{d}{dt}N_4^B = \underbrace{\lambda S_{N_4}}_{\text{Thymic output}} + \underbrace{\sum_\ell \left[\frac{1}{\alpha}\xi_2 N_4^\ell - \xi_1 N_4^B\right]}_{\text{Influx/Efflux}} - \underbrace{\sum_\ell k_1^\ell N_4^B \left(\frac{APC^\ell}{APC^\ell + hs_1^\ell}\right)}_{\text{Cytokine-mediated recruitment}} - \underbrace{\mu_8 N_4^B}_{\text{Natural death}}$$

BIE4C - E_4^B: The concentration of CD4+ effector T cells circulating in blood. These cells migrate from lymph nodes into the blood and leave the blood to the site of pulmonary infection. These cells also die in time.

$$\frac{d}{dt}E_4^B = \underbrace{\frac{\xi_3}{\alpha}\sum_\ell \left[E_4^\ell \left(1 - \frac{w_2^\ell M_I^\ell + M_A^\ell}{w_2^\ell M_I^\ell + M_A^\ell + hs_6^\ell}\right)\right]}_{\text{Lymph node migration}} - \underbrace{\xi_{E_4^B} E_4^B}_{\text{Recruitment to infection site}} - \underbrace{\mu_1 E_4^B}_{\text{Natural death}}$$

BICM4C - CM_4^B : CD4+ central memory T cells influx from / efflux to the lymph nodes. They are also recruited to the lymph nodes via cytokine-mediated recruitment. Here, we use APCs as a proxy for cytokine levels in the cells.

$$\frac{d}{dt}CM_4^B = \sum_\ell \left[\underbrace{\frac{1}{\alpha}\xi_5 CM_4^\ell - \xi_4 CM_4^B}_{\text{Influx/Efflux}} - \underbrace{k_8^\ell CM_4^B \left(\frac{APC^\ell}{APC^\ell + hs_8^\ell}\right)}_{\text{Cytokine-mediated recruitment to LNs}}\right]$$

BIEM4C -EM_4^B: CD4+ Effector memory cells efflux into the blood and are recruited to the site of pulmonary TB infection. They may die over time in the blood.

$$\frac{d}{dt}EM_4^B = \underbrace{\sum_{\ell}\left[\frac{\xi_6}{\alpha}EM_4^{\ell}\right]}_{\text{Lymph node migration}} - \underbrace{\xi_{EM_4^B}EM_4^B}_{\text{Recruitment to infection site}} - \underbrace{\mu_2 EM_4^B}_{\text{Natural death}} .$$

BIN8C - N_8^B: Naive CD8+ T cells are produced at a constant rate dependent upon the virtual host. They both influx from/efflux to lymph nodes and are recruited to lymph nodes via cytokine-mediated recruitment. Here, we use APCs as a proxy for the cytokine levels in the cells. Naive cells also die at a constant rate in blood.

$$\frac{d}{dt}N_8^B = \underbrace{\lambda S_{N_4}}_{\text{Thymic output}} + \underbrace{\sum_{\ell}\left[\frac{1}{\alpha}\xi_8 N_8^{\ell} - \xi_7 N_8^B\right]}_{\text{Influx/Efflux}} - \underbrace{\sum_{1}\left[k_{10}^{\ell}N_8^B\left(\frac{APC^{\ell}}{APC^{\ell} + hs_{10}^{\ell}}\right)\right]}_{\text{Cytokine-mediated recruitment to LNs}} - \underbrace{\mu_9 N_8^B}_{\text{Natural death}}$$

BIE8C - E_8^B: CD8+ Effector T cells efflux into the blood from the lymph nodes. From there, they either die or are recruited to the site of pulmonary infection.

$$\frac{d}{dt}E_8^B = \underbrace{\frac{\xi_9}{\alpha}\sum_{\ell}\left[E_8^{\ell}\left(1 - \frac{w_2^{\ell}M_I^{\ell} + M_A^{\ell}}{w_2^{\ell}M_I^{\ell} + M_A^{\ell} + hs_6^{\ell}}\right)\right]}_{\text{Lymph node migration}} - \underbrace{\xi_{E_8^B}E_8^B}_{\text{Recruitment to infection site}} - \underbrace{\mu_3 E_8^B}_{\text{Natural death}}$$

BICM8C - CM_8^B: CD8+ Central memory T cells can efflux to / influx from LNs. They are also recruited to the lymph nodes via cytokine-mediated recruitment. Here, we use APCs as a proxy for the cytokine levels in the cells.

$$\frac{d}{dt}CM_8^B = \sum_{\ell}\left[\underbrace{\frac{\xi_{11}}{\alpha}CM_8^{\ell} - \xi_{10}CM_8^B}_{\text{Influx / Efflux}} - \underbrace{k_{17}^{\ell}CM_8^B\left(\frac{APC^{\ell}}{APC^{\ell} + hs_{17}^{\ell}}\right)}_{\text{Cytokine-mediated recruitment to LNs}}\right]$$

BIEM8C - EM_8^B: CD8+ Effector memory cells efflux into the blood and are recruited to the site of pulmonary TB infection. They may die over time in the blood.

$$\frac{d}{dt}EM_8^B = \underbrace{\frac{\xi_{12}}{\alpha}\sum_{\ell}\left[EM_8^{\ell}\right]}_{\text{Lymph node migration}} - \underbrace{\xi_{EM_8^B}EM_8^B}_{\text{Recruitment to infection site}} - \underbrace{\mu_4 EM_8^B}_{\text{Natural death}}$$

BIN4Non - $N_4^{B,Non}$: Naive non-specific CD4+ T cells are produced by the thymus. They influx from / efflux to the lymph node and can be recruited to the lymph node by cytokines. Here, APCs serve as a proxy for cytokine levels in the LNs. Lastly, these cells may die in the blood.

$$\frac{d}{dt}N_4^{B,Non} = \underbrace{(1-\lambda)S_{N_4}}_{\text{Thymic output}} + \underbrace{\sum_{\ell}\left[\frac{1}{\alpha}\xi_2 N_4^{\ell,Non} - \xi_1 N_4^{B,Non}\right]}_{\text{Influx/Efflux}} - \underbrace{\sum_{\ell} k_1^{\ell} N_4^{B,Non}\left(\frac{APC^{\ell}}{APC^{\ell} + hs_1^{\ell}}\right)}_{\text{Cytokine-mediated recruitment}} - \underbrace{\mu_8 N_4^{B,Non}}_{\text{Natural death}}$$

BIE4Non - $E_4^{B,Non}$: Non-specific effector CD4+ T cells are produced by the thymus and die in time.

$$\frac{d}{dt}E_4^{B,Non} = S_{E_4^{Non}} - \mu_1 E_4^{B,Non}.$$

BICM4Non - $CM_4^{B,Non}$: Non-specific central memory CD4+ T cells efflux to / influx from lymph nodes and can be recruited to the lymph node by cytokines. Here, APCs serve as a proxy for cytokine levels in the LNs.

$$\frac{d}{dt}CM_4^{B,Non} = \sum_{\ell}\left[\underbrace{\frac{1}{\alpha}\xi_5 CM_4^{\ell} - \xi_4 CM_4^B}_{\text{Influx / Efflux}} - \underbrace{k_8^{\ell} CM_4^B\left(\frac{APC^{\ell}}{APC^{\ell} + hs_8^{\ell}}\right)}_{\text{Cytokine-mediated recruitment to LNs}}\right].$$

BIEM4Non -$EM_4^{B,Non}$: Non-specific effector memory CD4+ T cells are produced by the thymus and die in time.

$$\frac{d}{dt}E_4^{B,Non} = S_{EM_4^{Non}} - \mu_2 E_4^{B,Non}$$

BIN8Non - $N_8^{B,Non}$: Naive non-specific CD8+ T cells are produced by the thymus. They influx from / efflux to the lymph node and can be recruited to the lymph node by cytokines. Here, APCs serve as a proxy for cytokine levels in the LNs. Lastly, these cells may die in the blood.

$$\frac{d}{dt}N_8^{B,Non} = \underbrace{(1-\lambda)S_{N_8}}_{\text{Thymic output}} + \underbrace{\sum_{\ell}\left[\frac{1}{\alpha}\xi_8 N_8^{\ell,Non}\right]}_{\text{Influx/Efflux}} - \underbrace{\sum_{\ell} k_{10}^{\ell}\, N_8^{B,Non}\left(\frac{APC^{\ell}}{APC^{\ell}+hs_{10}^{\ell}}\right)}_{\text{Cytokine-mediated recruitment}} - \underbrace{\mu_9 N_8^{B,Non}}_{\text{Natural death}}.$$

BIE8Non - $E_8^{B,Non}$: Non-specific effector CD8+ T cells are produced by the thymus and die in time.

$$\frac{d}{dt}E_8^{B,Non} = S_{E_8^{Non}} - \mu_3 E_8^{B,Non}$$

BICM8Non - $CM_8^{B,Non}$: Non-specific central memory CD8+ T cells efflux to / influx from lymph nodes and can be recruited to the lymph node by cytokines. Here, APCs serve as a proxy for cytokine levels in the LNs.

$$\frac{d}{dt}CM_8^{B,Non} = \sum_{\ell}\left[\underbrace{\frac{1}{\alpha}\xi_{11}CM_8^{\ell} - \xi_{10}CM_8^{B}}_{\text{Influx / Efflux}} - \underbrace{k_{17}^{\ell}CM_8^{B}\left(\frac{APC^{\ell}}{APC^{\ell}+hs_{17}^{\ell}}\right)}_{\text{Cytokine-mediated recruitment to LNs}}\right].$$

BIEM8NC - $EM_8^{B,Non}$: Non-specific effector memory CD8+ T cells are produced by the thymus and die in time.

$$\frac{d}{dt}EM_8^{B,Non} = S_{EM_8^{Non}} - \mu_4 EM_8^{B,Non}$$

2 Lymph node equations

APC - APC^ℓ: Antigen presenting cells are not a state-variable solved for in this model. Rather, $APC^\ell(t) = APC^\ell/5$, where APC^ℓ is a function in time that we generated by our previously-established model of pulmonary TB infection, *HostSim.* The specific trajectory was the same for all lymph nodes and hosts, and was taken from a virtual host with latent pulmonary infection or a virtual host with active pulmonary infection generated by the version of *HostSim* presented in (1). **Figure 2A-B** in main text shows the APC trajectory in time.

LnN4C - N_4^ℓ: Naive CD4+ T cells both influx from / efflux to the blood, are recruited to the LN from blood by cytokine-driven recruitment and differentiate into precursor CD4+ T cells.

$$\frac{d}{dt}N_4^\ell = \underbrace{\alpha\xi_1 N_4^B - \xi_2 N_4^\ell}_{\text{Influx/Efflux}} + \underbrace{\alpha k_1^\ell N_4^B\left(\frac{APC^\ell}{APC^\ell + hs_1^\ell}\right)}_{\text{Cytokine-mediated recruitment}} - \underbrace{k_2^\ell APC^\ell N_4^\ell}_{\text{Differentiation into } P_4^\ell}$$

LnP4C - P_4^ℓ: Precursor CD4+ T cells proliferate based on presence of APC up to a carrying capacity ρ^ℓ. They differentiate from N_4^ℓ; and CM_4^ℓ, and differentiate into E_4^ℓ and CM_4^ℓ. They can also die over time in the lymph nodes.

$$\frac{d}{dt}P_4^\ell = \underbrace{APC^\ell\left(k_2^\ell N_4^\ell + k_3^\ell CM_4^\ell\right)}_{\text{Differentiation from } N_4^\ell \text{ and } CM_4^\ell} + \underbrace{k_4^\ell P_4^\ell\left(1 - \frac{P_4^\ell}{\rho_1^\ell}\right)\left(\frac{APC^\ell}{APC^\ell + hs_4^\ell}\right)}_{\text{Proliferation}} - \underbrace{k_6^\ell P_4^\ell\left(\frac{APC^\ell}{APC^\ell + hs_5^\ell}\right)}_{\text{Cytokine-mediated differentiation into } E_4^\ell}$$

$$- \underbrace{k_6^\ell P_4^\ell\left(1 - \frac{APC^\ell}{APC^\ell + hs_5^\ell}\right)}_{\text{Cytokine-mediated differentiation into } CM_4^\ell} - \underbrace{\mu_6 P_4^\ell}_{\text{Natural Death}}$$

LnE4C - E_4^ℓ: CD4+ Effector T cells in LNs are generated via differentiation of P_4^ℓ, and can differentiate into EM_4^ℓ. They can change compartments by either effluxing to blood or being recruited to the lymph-node granulomas.

$$\frac{d}{dt}E_4^\ell = \underbrace{k_6^\ell P_4^\ell\left(\frac{APC^\ell}{APC^\ell + hs_5^\ell}\right)}_{\text{Cytokine-mediated differentiation from } P_4^\ell} \underbrace{-\,\xi_3 E_4^\ell\left(1 - \frac{w_2^\ell M_I^\ell + M_A^\ell}{w_2^\ell M_I^\ell + M_A^\ell + hs_6^\ell}\right)}_{\text{Lymph node migration to blood}} \underbrace{-\,\xi_3 E_4^\ell \frac{w_2^\ell M_I^\ell + M_A^\ell}{w_2^\ell M_I^\ell + M_A^\ell + hs_6^\ell}}_{\text{Recruitment to LN granuloma}}$$
$$- \underbrace{k_7^\ell E_4^\ell}_{\text{Differentiation into } EM_4^\ell}$$

LnCM4C - CM_4^ℓ: CD4+ Central memory T cells can influx from the blood. Cytokine-mediated recruitment also pulls these cells from blood, where APCs serve as a proxy for cytokines in the LN. Central memory cells can also proliferate from precursor cells (P_4^ℓ) in the absence of APCs. Central memory cells can efflux from the blood or differentiate into P_4^ℓ in the presence of APCs.

$$\frac{d}{dt}CM_4^\ell = \underbrace{\alpha\xi_4 CM_4^B - \xi_5 CM_4^\ell}_{\text{Influx/Efflux}} + \underbrace{\alpha k_8^\ell CM_4^B\left(\frac{APC^\ell}{APC^\ell + hs_8^\ell}\right)}_{\text{Cytokine-mediated recruitment to LNs}} + \underbrace{k_6^\ell P_4^\ell\left(1 - \frac{APC^\ell}{APC^\ell + hs_5^\ell}\right)}_{\text{Cytokine-mediated differentiation from } P_4^\ell}$$
$$- \underbrace{APC^\ell k_3^\ell CM_4^\ell}_{\text{Differentiation to } P_4^\ell}$$

LnEM4C - EM_4^ℓ: CD4+ Effector memory T cells in the LNs differentiate from effector cells (E_4^ℓ) and efflux to blood.

$$\frac{d}{dt}EM_4^\ell = \underbrace{k_7^\ell E_4^\ell}_{\text{Differentiation from } E_4^\ell} - \underbrace{\xi_6 EM_4^\ell}_{\text{Efflux to blood}}$$

LnN8 -N_8^ℓ: Naive CD8+ T cells influx from / efflux to blood, and also are recruited from blood with cytokine-mediation. Here, APCs in the LN serve as a proxy for cytokines in the LN. CD8+ T cells can also differentiate into precursor CD8+ T cells (P_8^ℓ) in the presence of CD4+ effector (E_4^ℓ) or precursor (P_4^ℓ) T cells.

$$\frac{d}{dt}N_8^\ell = \underbrace{\alpha\xi_7 N_8^B - \xi_8 N_8^\ell}_{\text{Influx / Efflux}} + \underbrace{\alpha k_{10}^\ell N_8^B\left(\frac{APC^\ell}{APC^\ell + hs_{10}^\ell}\right)}_{\text{Cytokine-mediated recruitment}} - \underbrace{k_{11}^\ell APC^\ell N_8^\ell\left(\frac{E_4^\ell + w_{P_4}P_4^\ell}{E_4^\ell + w_{P_4}P_4^\ell + hs_{11}^\ell}\right)}_{\text{Differentiation into } P_8^\ell}$$

LnP8C - P_8^ℓ: Precursor CD8+ T cells in the LN are differentiated from CD8+ Naive (N_8^ℓ) or central memory (CM_8^ℓ) T cells in the presence of APCs. They can proliferate up to a carrying capacity ρ_1 in the presence of APCs. Precursor cells can differentiate into effector cells (E_8^ℓ) and central memory cells (CM_8^ℓ). Precursor cells can die in the lymph node.

$$\frac{d}{dt}P_8^\ell = \underbrace{k_{11}^\ell APC^\ell N_8^\ell\left(\frac{E_4^\ell + w_{P_4}P_4^\ell}{E_4^\ell + w_{P_4}P_4^\ell + hs_{11}^\ell}\right)}_{\text{Differentiation from } N_8^\ell} + \underbrace{k_{13}^\ell P_8^\ell\left(1 - \frac{P_8^\ell}{\rho_1}\right)\left(\frac{APC^\ell}{APC^\ell + hs_{13}^\ell}\right)}_{\text{Proliferation}} + \underbrace{k_{12}^\ell CM_8^\ell APC^\ell}_{\text{Differentiation from } CM_8^\ell}$$
$$- \underbrace{k_{15}^\ell P_8^\ell\left(1 - \frac{APC^\ell}{APC^\ell + hs_{14}^\ell}\right)}_{\text{Differentiation to } CM_8^\ell} - \underbrace{k_{14}^\ell P_8^\ell\left(\frac{APC^\ell}{APC^\ell + hs_{14}^\ell}\right)}_{\text{Differentiation to } E_8^\ell} - \underbrace{\mu_7 P_8^\ell}_{\text{Natural death}}$$

LnE8C - E_8^ℓ: Effector CD8+ T cells can be generated in the lymph node by differentiation from precursor CD8+ T cells (P_8^ℓ). They can also migrate to blood, differentiate into effector memory (EM_8^ℓ) cells, or be recruited to the lymph-node granuloma.

$$\frac{d}{dt}E_8^\ell = \underbrace{k_{14}^\ell P_8^\ell\left(\frac{APC^\ell}{APC^\ell + hs_{14}^\ell}\right)}_{\text{Differentiation from} P_8^\ell} - \underbrace{\xi_9 E_8^\ell\left(1 - \frac{w_2^\ell M_I^\ell + M_A^\ell}{w_2^\ell M_I^\ell + M_A^\ell + hs_6^\ell}\right)}_{\text{Migration to blood}} - \underbrace{\xi_9 E_8^\ell\left(\frac{w_2^\ell M_I^\ell + M_A^\ell}{w_2^\ell M_I^\ell + M_A^\ell + hs_6^\ell}\right)}_{\text{Recruitment to LN granuloma}}$$
$$- \underbrace{k_{16} E_8^\ell}_{\text{Differentiation into } EM_8^\ell}$$

LnCM8C - CM_8^ℓ: CD8+ central memory T cells both influx from and efflux to the blood. They also can be recruited from the blood, mediated by cytokines. Here, APCs are a proxy for cytokine levels in the LN. Central memory cells can also differentiate into or from precursor cells (P_8^ℓ), depending on the presence of APCs in the LN.

$$\frac{d}{dt}CM_8^\ell = \underbrace{\alpha\xi_{10}CM_8^B - \xi_{11}CM_8^\ell}_{\text{Influx/Efflux}} + \underbrace{\alpha k_{17}^\ell CM_8^B\left(\frac{APC^\ell}{APC^\ell + hs_{17}^\ell}\right)}_{\text{Cytokine-mediated recruitment to LNs}} + \underbrace{k_{15}^\ell P_8^\ell\left(1 - \frac{APC^\ell}{APC^\ell + hs_{14}^\ell}\right)}_{\text{Cytokine-mediated differentiation from } P_8^\ell}$$
$$- \underbrace{APC^\ell k_{12}^\ell CM_8^\ell}_{\text{Differentiation to } P_8^\ell}$$

LnEM8C - EM_8^ℓ: CD8+ Effector memory T cells in the LNs differentiate from effector cells (E_8^ℓ) and efflux to blood.

$$\frac{d}{dt}EM_4^\ell = \underbrace{k_{16}^\ell E_8^\ell}_{\text{Differentiation from } E_8^\ell} - \underbrace{\xi_{12}EM_8^\ell}_{\text{Efflux to blood}}$$

LnN4Non - $N_4^{\ell,Non}$: Non-specific naive CD4+ T cells are recruited to LNs by cytokines (with APCs as a proxy for cytokine levels). These cells also influx from / efflux to the blood.

$$\frac{d}{dt}N_4^{\ell,Non} = \underbrace{\alpha\xi_1 N_4^{B,Non} - \xi_2 N_4^{\ell,Non}}_{\text{Influx/Efflux}} + \underbrace{\alpha k_1^\ell N_4^{B,Non}\left(\frac{APC^\ell}{APC^\ell + hs_1^\ell}\right)}_{\text{Cytokine-mediated recruitment}}$$

LnCM4Non - $CM_4^{\ell,Non}$: Non-specific central-memory CD4+ T cells are recruited to LNs by cytokines (with APCs as a proxy for cytokine levels). These cells also influx from / efflux to the blood.

$$\frac{d}{dt}CM_4^{\ell,Non} = \underbrace{\alpha\xi_4 CM_4^{B,Non} - \xi_5 CM_4^{\ell,Non}}_{\text{Influx/Efflux}} + \underbrace{\alpha k_8^\ell CM_4^{B,Non}\left(\frac{APC^\ell}{APC^\ell + hs_8^\ell}\right)}_{\text{Cytokine-mediated recruitment}}$$

LnN8Non - $N_8^{\ell,Non}$: Non-specific naive CD8+ T cells are recruited to LNs by cytokines (with APCs as a proxy for cytokine levels). These cells also influx from / efflux to the blood.

$$\frac{d}{dt}N_8^{\ell,Non} = \underbrace{\alpha\xi_7 N_8^{B,Non} - \xi_8 N_8^{\ell,Non}}_{\text{Influx/Efflux}} + \underbrace{\alpha k_{10}^{\ell} N_8^{B,Non}\left(\frac{APC^{\ell}}{APC^{\ell} + hs_{10}^{\ell}}\right)}_{\text{Cytokine-mediated recruitment}}$$

LnCM8Non - $CM_8^{\ell,Non}$: Non-specific central memory CD8+ T cells are recruited to LNs by cytokines (with APCs as a proxy for cytokine levels). These cells also influx from / efflux to the blood.

$$\frac{d}{dt}CM_8^{\ell,\mathrm{Non}} = \underbrace{\alpha\xi_{10} CM_8^{B,Non} - \xi_{11} CM_8^{\ell,Non}}_{\text{Influx/Efflux}} + \underbrace{\alpha k_{17}^{\ell} CM_8^{B,Non}\left(\frac{APC^{\ell}}{APC^{\ell} + hs_{17}^{\ell}}\right)}_{\text{Cytokine-mediated recruitment}}$$

3 LN granuloma equations

Although the below equations may be defined for all lymph nodes, in our study we only instantiated nonzero bacteria in lymph nodes 1 and 2 (i.e.$\ell = 1{,}2$). These equations were based on the lung granuloma equations from *HostSim* (1,2).

LnMR - M_R^ℓ: Resting macrophages can be recruited based on cytokine signaling in the granuloma, where a weighted sum of M_A^ℓ; and M_I^ℓ; is used as a proxy for cytokine signaling in the granuloma. The recruitment can occur up to a carrying capacity. Resting macrophages become infected by Mtb (i.e. become M_I^ℓ) or are activated. Proxies for signals to activate macrophages include counts of extracellular bacteria (B_E^ℓ) and granuloma-involved CD4+ T-cells G_4^ℓ. Resting macrophages can also die over time.

$$\frac{d}{dt}M_R^\ell = \underbrace{\alpha_{4a}^\ell\left(M_A^\ell + w_2^\ell M_I^\ell\right)\left(1 - \frac{M_R^\ell}{n_2^\ell}\right)}_{\text{Macrohpage-driven recruitment}} - \underbrace{k_2^\ell M_R^\ell\left(\frac{B_E^\ell}{B_E^\ell + c_9^\ell}\right)}_{\text{Macrophage infection}} - \underbrace{k_3^\ell M_R^\ell\left(\frac{B_E^\ell + w_1^\ell B_I^\ell}{B_E^\ell + w_1^\ell B_I^\ell + c_8^\ell}\right)\left(\frac{G_4^\ell}{G_4^\ell + hs_4^\ell}\right)}_{\text{Activation of macrophages}}$$
$$- \underbrace{\mu_{M_R}^\ell M_R^\ell}_{\text{Natural death}}$$

LnMI - M_I^ℓ: Infected macrophages are generated by the infection of resting macrophages (M_R^ℓ). Infected macrophages can burst when more than n_1^ℓ; bacteria fill them (on average), converting $\frac{B_I^\ell}{M_I^\ell}$ intracellular bacteria into extracellular bacteria (B_E^ℓ) and killing the macrophage. Infected macrophages can be killed by T cell driven apoptosis by CD4+ and CD8+ T cells that have been recruited to the granuloma (G_4^ℓ and G_8^ℓ). Infected macrophages can die naturally over time.

$$\frac{d}{dt}M_I^\ell = \underbrace{k_2^\ell M_R^\ell\left(\frac{B_E^\ell}{B_E^\ell + c_9^\ell}\right)}_{\text{Macrophage infection}} - \underbrace{k_{17}^\ell M_I^\ell\left(\frac{\left(B_I^\ell\right)^{p^\ell}}{\left(B_I^\ell\right)^{p^\ell} + \left(n_1^\ell M_I^\ell\right)^{p^\ell}}\right)}_{\text{Macrophage bursting}}$$

$$\underbrace{- k_{52}^\ell M_I^\ell\left(\frac{G_8^\ell\left(\frac{G_4^\ell}{G_4^\ell + c_{E_4}^\ell}\right) + w_1^\ell G_4^\ell}{G_8^\ell\left(\frac{G_4^\ell}{G_4^\ell + c_{E_4}^{1}}\right) + w_1^\ell G_4^\ell + M_I^\ell c_{52}^\ell}\right)}_{\text{T-cell driven apoptosis of infected macrophages}} - \underbrace{\mu_{M_I}^\ell M_I^\ell}_{\text{Natural death}}$$

LnMA – M_A^ℓ: Activated macrophages are the result of activation of resting macrophages (M_R^ℓ) and die naturally in time

$$\frac{d}{dt}M_A^\ell = \underbrace{k_3^\ell M_R^\ell\left(\frac{B_E^\ell + w_1^\ell B_I^\ell}{B_E^\ell + w_1^\ell B_I^\ell + c_8^\ell}\right)\left(\frac{G_4^\ell}{G_4^\ell + hs_4^\ell}\right)}_{\text{Activation of macrophages}} - \underbrace{\mu_{M_A}^\ell M_A^\ell}_{\text{Natural death}}$$

LnBI – B_I^ℓ: Intracellular Mtb replicate within infected macrophages (M_I^ℓ) up to a carrying capacity. Mtb also become B_I^ℓ once an extracellular bacterium (B_E^ℓ) is internalized by a macrophage (M_R^ℓ). We assume that an average resting macrophage picks up a number of extracellular Mtb equal to half of the single-macrophage intracellular carrying capacity of Mtb (N_1^ℓ). When infected macrophages naturally die, a fraction of them (λ_{surv}) survive to become extracellular bacteria (B_E^ℓ); when an infected macrophage bursts, the maximum number of bacteria that can fit within a macrophage (N_1^ℓ) are released. If an infected macrophage is killed via T-cell driven apoptosis, the current average number of bacteria per infected macrophage ($\frac{B_I^\ell}{\mathrm{M}_\mathrm{I}^\ell}$) also die. Intracellular bacteria can die in time, albeit very slowly.

$$\frac{d}{dt}B_I^\ell = \underbrace{\alpha_{19}^\ell B_I^\ell\left(1-\frac{B_I^\ell/M_I^\ell}{N_1^\ell}\right)}_{\text{Intracellular replication}} + \underbrace{k_2^\ell\frac{N_1^\ell}{2}M_R^\ell\left(\frac{B_E^\ell}{B_E^\ell+c_9^\ell}\right)}_{\text{Macrophage infection}} - \underbrace{k_{17}^\ell N_1^\ell M_I^\ell\left(\frac{\left(B_I^\ell\right)^{p^\ell}}{\left(B_I^\ell\right)^{p^\ell}+\left(n_1^\ell M_I^\ell\right)^{p^\ell}}\right)}_{\text{Macrophage bursting}}$$

$$- \underbrace{k_{52}^\ell M_I^\ell\frac{B_I^\ell}{M_I^\ell}\left(\frac{G_8^\ell\left(\frac{G_4^\ell}{G_4^\ell+c_{E_4}^\ell}\right)+w_1^\ell G_4^\ell}{G_8^\ell\left(\frac{G_4^\ell}{G_4^\ell+c_{E_4}^\ell}\right)+w_1^\ell G_4^\ell+M_I^\ell c_{52}^\ell}\right)}_{\text{T-cell driven apoptosis of infected macrophages}} - \underbrace{\mu_{B_I}^\ell B_I^\ell}_{\text{Natural death of } B_I}$$

$$- \underbrace{\mu_{M_I}^\ell\frac{B_I^\ell}{M_I^\ell}M_I^\ell}_{\text{Release of } B_I \text{ by naturally dying } M_I}$$

LnBE – B_E^ℓ: Extracellular bacteria replicate within the granuloma up to a carrying capacity N_3^ℓ. When infected macrophages naturally die, a fraction of them (λ_{surv}^ℓ) survive to become extracellular bacteria (B_E^ℓ). When infected macrophages are killed by T-cell driven apoptosis, a fraction of them (N_{fracc}^ℓ) survive to become extracellular bacteria (B_E^ℓ).

$$\frac{d}{dt}B_E^\ell = \underbrace{\alpha_{20}^\ell B_E^\ell\left(1-\frac{B_E^\ell}{N_3^\ell}\right)}_{\text{Extracellular Mtb replication}} + \underbrace{\mu_{M_I}^\ell \lambda_{surv}^\ell B_I^\ell}_{\text{Release of } B_I \text{ by naturally dying } M_I} + \underbrace{k_{17}^\ell N_1^\ell M_I^\ell\left(\frac{\left(B_I^\ell\right)^{p^\ell}}{\left(B_I^\ell\right)^{p^\ell}+\left(n_1^\ell M_I^\ell\right)^{p^\ell}}\right)}_{\text{Macrophage bursting}}$$

$$+\underbrace{k_{52}^\ell N_{fracc}^\ell B_I^\ell\left(\frac{G_8^\ell\left(\frac{G_4^\ell}{G_4^\ell+c_{E_4}^\ell}\right)+w_1^\ell G_4^\ell}{G_8^\ell\left(\frac{G_4^\ell}{G_4^\ell+c_{E_4}^\ell}\right)+w_1^\ell G_4^\ell+M_I^\ell c_{52}^\ell}\right)}_{\text{T-cell driven apoptosis of infected macrophages}} - \underbrace{k_2^\ell \frac{N_1^\ell}{2} M_R^\ell\left(\frac{B_E^\ell}{B_E^\ell+c_9^\ell}\right)}_{\text{Macrophage infection}}$$

$$-\underbrace{k_{15}^\ell M_A^\ell B_E^\ell}_{\text{Activated macrophage killing of } B_E} - \underbrace{k_{18}^\ell M_R^\ell B_E^\ell}_{\text{Resting macrophage killing of } B_E} - \underbrace{\mu_{B_E}^\ell B_E^\ell}_{\text{Natural death}}$$

LnG4C – G_4^ℓ: Granuloma-involved CD4+ T-cells are recruited to the granuloma based on macrophage saturation and can proliferate within granulomas in the presence of infected macrophages.

$$\frac{d}{dt}G_4^\ell = \underbrace{\xi_3 E_4^\ell \frac{w_2^\ell M_I^\ell + M_A^\ell}{w_2^\ell M_I^\ell + M_A^\ell + hs_6^\ell}}_{\text{Recruitment from LN}} + \underbrace{k_9^\ell G_4^\ell \left(\frac{\rho_2^\ell}{G_4^\ell + \rho_2^\ell}\right)\left(\frac{M_I^\ell}{M_I^\ell + hs_6^\ell}\right)}_{\text{Macrophage-mediated proliferation}}$$

LnG8C – G_8^ℓ: Granuloma-involved CD8+ T-cells are recruited to the granuloma based on macrophage saturation and can proliferate within granulomas in the presence of infected macrophages.

$$\frac{d}{dt}G_8^\ell = \underbrace{\xi_9 E_8^\ell \frac{w_2^\ell M_I^\ell + M_A^\ell}{w_2^\ell M_I^\ell + M_A^\ell + hs_6^\ell}}_{\text{Recruitment from LN}} + \underbrace{k_{19}^\ell G_8^\ell \left(\frac{\rho_3^\ell}{G_8^\ell + \rho_3^\ell}\right)\left(\frac{M_I^\ell}{M_I^\ell + hs_6^\ell}\right)}_{\text{Macrophage-mediated proliferation}}$$
