# Supplementary material for: Distinguishing multiple roles of T cell and macrophage involvement in determining lymph node fates during Mycobacterium tuberculosis infection
Source: PLoS Comput Biol. 2025 May 7;21(5):e1013033. doi: 10.1371/journal.pcbi.1013033 (PMC12084042; doi:10.1371/journal.pcbi.1013033)
Supplement: S2 Appendix — This appendix provides a complete list of model parameters for equations given in S1 Appendix. Table A details blood parameters. Table B details lymph node parameters. Table C details lymph node granuloma parameters. Column 1 shows the 5 searchable name of each parameter. Bl refers to blood. Column 2 shows the symbol used in the equations. Column 3 givens a 6 description of the parameter. The last 3 columns refer to the uncertainty analysis parameter distributions and the range values of minimum and maximum. (PDF) [file pcbi.1013033.s002.pdf]

S2 Appendix for

Distinguishing multiple roles of T cells and macrophage involvement in determining lymph node fates during *Mycobacterium tuberculosis* infection

K.C. Krupinsky, C.T. Michael, P. Nanda, J. Mattila, D. Kirschner

This appendix provides a complete list of model parameters is given for the equations given in **S1 Appendix**. Column 1 shows the searchable name of each parameter. Bl refers to blood. Column 2 shows the symbol used in the equations. Column 3 givens a description of the parameter. CM4 refers to CD4+ central memory T cells, CM8 refers to CD8+ central memory T cells, E4 refers to CD4+ effector T cells, E8 refers to CD8+ effector T cells, EM4 refers to CD4+ effector memory T cells, EM8 refers to CD8+ effector memory T cells, N4 refers to CD4+ naïve T cells, N8 refers to CD8+ naïve T cells, P8 refers to CD8+ precursor T cells, P4 refers to CD4+ precursor T cells, MR refers to resting macrophages, MI refers to infected macrophages, MA refers to activated macrophages, BI refers to intracellular Mtb bacteria, BE refers to extracellular Mtb bacteria, G4 refers to granuloma-associated CD4+ T cells, and G8 refers to CD8+ T cells. The last 3 columns refer to the uncertainty analysis parameter distributions and the range of values of minimum and maximum.

SUPPLEMENTARY TABLE A. Blood Parameters						
**Name**	**Symbol**	**Description**	**Units**	**Distribution**	**Minimum**	**Maximum**
BlCM4	CM_4^B	blood concentration of CM4	cells/uL	uniform	5.94	452
BlCM4CogFraction	$CogFrac_{CM_4^B}$	Mtb-specific fraction of blood CM4	-	-	0	0
BlCM8	CM_8^B	blood concentration of CM8	cells/uL	uniform	5.94	452
BlCM8CogFraction	$CogFrac_{CM_8^B}$	Mtb-specific fraction of blood CM8	-	-	0	0
BlE4	E_4^B	blood concentration of E4	cells/uL	uniform	30.6	329
BlE8	E_8^B	blood concentration of E8	cells/uL	uniform	30.6	329
BlEM4	EM_4^B	blood concentration of EM4	cells/uL	uniform	31.4	320
BlEM4CogFraction	$CogFrac_{EM_4^B}$	Mtb-specific fraction of blood EM4	-	-	0	0
BlEM8	EM_8^B	blood concentration of EM8	cells/uL	uniform	31.4	320
BlEM8CogFraction	$CogFrac_{EM_8^B}$	Mtb-specific fraction of blood EM8	-	-	0	0
BlN4	N_4^B	blood concentration of total N4	cells/uL	uniform	6.51	858
BlN8	N_8^B	blood concentration of N8	cells/uL	uniform	6.51	858
alpha	α	total volume of blood within a host	uL	-	360,000	360,000

host_Ln	Ln_{host}	number of host LNs	count	uniform	500	700
lambda	λ	frequency of Mtb-specific N4	cells/uL	uniform	1e-04	0.1
lnDistPercent	$Ln_{DistPercent}$	ratio of lymph node to blood cells	-	uniform	0.05	0.1
semnc4	$\lambda S_{EM_4^{NC}}$	EM4 output from thymus	count/day	computed	-	-
semnc8	$\lambda S_{EM_8^{NC}}$	EM8N output from thymus	count/day	computed	-	-
senc4	$\lambda S_{E_4^{NC}}$	E4N output from thymus	count/day	computed	-	-
senc8	$\lambda S_{E_8^{NC}}$	E8N output from thymus	count/day	computed	-	-
sn4	λS_{N_4}	N4 output from thymus	count/day	computed	-	-
sn8	λS_{N_8}	N8 output from thymus	count/day	computed	-	-
wp4	w_{P_4}	contribution of Mtb-specific P4 to Mtb-specific N8 priming	-	-	0.736	0.736
xi1	ξ_1	influx of N4	1/day	computed	-	-
xi10	ξ_{10}	influx of CM8	1/day	computed	-	-
xi11	ξ_{11}	efflux of CM8	1/day	uniform	1.13	2.22
xi12	ξ_{12}	efflux of EM8	1/day	log-uniform	1.22e-06	0.252
xi2	ξ_2	efflux of N4	1/day	uniform	1.2	2.51

xi4	ξ_4	influx of CM4	1/day	computed	-	-
xi5	ξ_5	efflux of CM8	1/day	uniform	1.07	2.92
xi6	ξ_6	efflux of EM4	1/day	log-uniform	0.00157	29.2
xi7	ξ_7	influx of N8	1/day	computed	-	-
xi8	ξ_8	efflux of N8	1/day	uniform	1.16	5.21
xi9	ξ_9	efflux of E8	1/day	log-uniform	1.87e-06	0.249
mu1	μ_1	death of E4	1/day	-	0.2	0.2
mu2	μ_2	death of EM4	1/day	uniform	0.00137	0.00274
mu3	μ_3	death of E8	1/day	-	0.2	0.2
mu4	μ_4	death of EM8	1/day	uniform	0.00137	0.00274
mu8	μ_8	death of N4	1/day	uniform	0.003	3
mu9	μ_9	death of N8	1/day	log-uniform	5e-04	0.05
xiE4C	$\xi_{E_4^C}$	trafficking to the lung of Mtb-specific E4	1/day	uniform	0.1	0.9
xiE8C	$\xi_{E_8^C}$	trafficking to the lung of Mtb-specific E8	1/day	uniform	0.1	0.9
xiEM4C	$\xi_{EM_4^C}$	trafficking to the lung of Mtb-specific EM4	1/day	uniform	0.1	0.9

xiEM8C	$\xi_{EM_8^C}$	trafficking to the lung of Mtb-specific EM8	1/day	uniform	0.1	0.9

SUPPLEMENTARY TABLE B. Lymph Node Parameters

Name	Symbol	Description	Units	Distribution	Minimum	Maximum
hs11	hs_{11}	half-sat of N8 priming	count	uniform	14.3	473
hs14	hs_{14}	half-sat of P8 differentiation	count	uniform	16,900	41,100
k11	k_{11}	priming of N8	1/day	uniform	0.25	0.958
k14	k_{14}	differentiation of P8 to E8	1/day	uniform	0.294	1.53
k15	k_{15}	differentiation of P8 to CM8	1/day	uniform	0.0606	1.28
k16	k_{16}	differentiation of P8 to EM8	1/day	uniform	0.618	1.24
k2	k_2	priming of N4	1/day	uniform	0.24	1.63
k6	k_6	differentiation of P4 to CM4	1/day	uniform	0.00279	1.32
hs7	hs_7	half-sat of macrophages on T-cell efflux	count	log-uniform	200	9,000
mu6	μ_6	death of P4	1/day	-	5e-04	5e-04
mu7	μ_7	death of P8	1/day	-	0.015	0.015

hs13	hs_{13}	half-sat of P8 proliferation	count	uniform	9,270	79,700
hs4	hs_4	half-sat of P4 proliferation	count	uniform	4,060	28,400
k13	k_{13}	proliferation of P8	1/day	uniform	2.09	4.63
k4	k_4	proliferation of P4	1/day	uniform	2.08	4.73
rho1	ρ_1	carrying capacity of P4 proliferation	count	log-uniform	495	14,700,000
k12	k_{12}	reactivation of CM8	1/day	uniform	0.00154	0.0954
k3	k_3	reactivation of CM4	1/day	uniform	0.0319	0.0565
hs1	hs_1	half-sat of N4 recruitment	count	uniform	2,490	22,800
hs10	hs_{10}	half-sat of N8 recruitment	count	uniform	2,980	10,800
hs17	hs_{17}	half-sat of CM8 recruitment	count	uniform	89.3	10,700
hs8	hs_8	half-sat of CM4 recruitment	count	uniform	4,130	8,010
k1	k_1	recruitment of N4	1/day	uniform	0.388	1.32
k10	k_{10}	recruitment of N8	1/day	uniform	0.253	0.307
k17	k_{17}	recruitment of CM8	1/day	log-uniform	3.15e-06	0.6
k8	k_8	recruitment of CM4	1/day	uniform	0.038	0.0679

SUPPLEMENTARY TABLE C. Lymph Node Granuloma Parameters						
**Name**	**Symbol**	**Description**	**Units**	**Distribution**	**Minimum**	**Maximum**
frac	λ_{surv}	fraction of BI released by natural MI death	-	uniform	0.000941	0.00111
k15	k_{15}	MA-mediated death of BE	1/day	uniform	0.00691	0.103
muBE	μ_{B_E}	natural death of BE	1/day	uniform	4.11e-09	7.41e-09
muBI	μ_{B_I}	natural death of BI	1/day	uniform	5.95e-05	9.04e-05
nfracc	N_{frac_c}	fraction of BI released by T-cell killing of MI	-	uniform	0.307	0.789
alpha19	α_{19}	growth of BI	1/day	log-uniform	0.0867	1.32
alpha20	α_{20}	growth of BE	1/day	uniform	0.0253	0.943
n3	n_3	carrying capacity of BE within LN granuloma	count	uniform	692,000	9,030,000
hs16	hs_{16}	half-sat of G8C proliferation	count	uniform	2,000	9,000
hs6	hs_6	half-sat of G4C proliferation	count	uniform	2,000	9,000
k19	k_{19}	proliferation of G8	1/day	log-uniform	0.0108	5.73
k9	k_9	proliferation of G4	1/day	log-uniform	0.0911	5.73

rho2	ρ_2	carrying capacity of G4 proliferation	count	log-uniform	495	1,470,000
rho3	ρ_3	carrying capacity of G8 proliferation	count	log-uniform	495	1,470,000
xi9	ξ_9	recruitment of G8	1/day	uniform	18.7	24.5
c8	c_8	half-sat of BE and BI on MR activation	count	uniform	3,640	8,090
hs4	hs_4	half-sat of G4 on MR activation	count	log-uniform	84.8	51,000
k3	k_3	activation of MR	1/day	uniform	0.0344	0.0455
w1	w_1	contribution of BI to MR activation	-	uniform	0.26	0.563
c52	c_{52}	half-sat of T-cell induced MI apoptosis	count	log-uniform	51,800	1,620,000
cLnE4C	$c_{E_{4C}^{Ln}}$	half-sat of G4 on MI apoptosis	count	log-uniform	45.3	1,770,000
k17	k_{17}	bursting of MI	1/day	uniform	0.0884	0.238
k52	k_{52}	T-cell mediated death of MI	1/day	log-uniform	0.00955	3.91
muMA	μ_{MR}	natural death of MA	1/day	uniform	0.05	0.1
muMI	μ_{MI}	natural death of MI	1/day	uniform	0.00297	0.0038
muMR	μ_{MA}	natural death of MR	1/day	uniform	0.00443	0.0057

n1	n_1	carrying capacity of bacteria within MI	count	log-uniform	0.603	2710
c9	c_9	half-sat of BE on MR infection	count	uniform	1,310	9,190
k2	k_2	infection of MR	1/day	uniform	0.136	2.31
alpha4a	α_{4a}	recruitment of MR	1/day	uniform	0.0852	0.46
n2	n_2	carrying capacity of MR	count	uniform	8,920,000	1.2e+07
w2	w_2	contribution of MI to MR recruitment	-	uniform	0.357	1.17
