# Supplementary material for: Distinguishing multiple roles of T cell and macrophage involvement in determining lymph node fates during Mycobacterium tuberculosis infection
Source: PLoS Comput Biol. 2025 May 7;21(5):e1013033. doi: 10.1371/journal.pcbi.1013033 (PMC12084042; doi:10.1371/journal.pcbi.1013033)
Supplement: S1 Text — This document details pulmonary status of actively infected host (Fig A), model blood (Fig B) and negative control calibration (Fig C), and actively infected host analyses that parallel the LTBI host presented in the manuscript (S1-S5 Figs). (PDF) [file pcbi.1013033.s003.pdf]

S1 Text for

Distinguishing multiple roles of T cells and macrophage involvement in determining lymph node fates during *Mycobacterium tuberculosis* infection

K.C. Krupinsky, C.T. Michael, P. Nanda, J. Mattila, D. Kirschner

The material in this supplement shows a parallel case of results to the main body of the paper for active pulmonary infection hosts in contrast to LTBI hosts that were presented. Additionally, it provides simulation data on the blood compartment and negative control (healthy) cases for both LTBI hosts and active pulmonary infection hosts.

We repeat the same cases as show in the main body of the paper within virtual hosts with active pulmonary infection and find similar results (**S1-S5 Figs**). Hosts with active pulmonary infection show a maintenance of T cells throughout an entire simulation period (rather than returning to pre-infection levels seen within LTBI hosts). This is attributable to continued APC stimulation in hosts with active pulmonary infection that is not seen within LTBI hosts **(Fig 3A-B).**

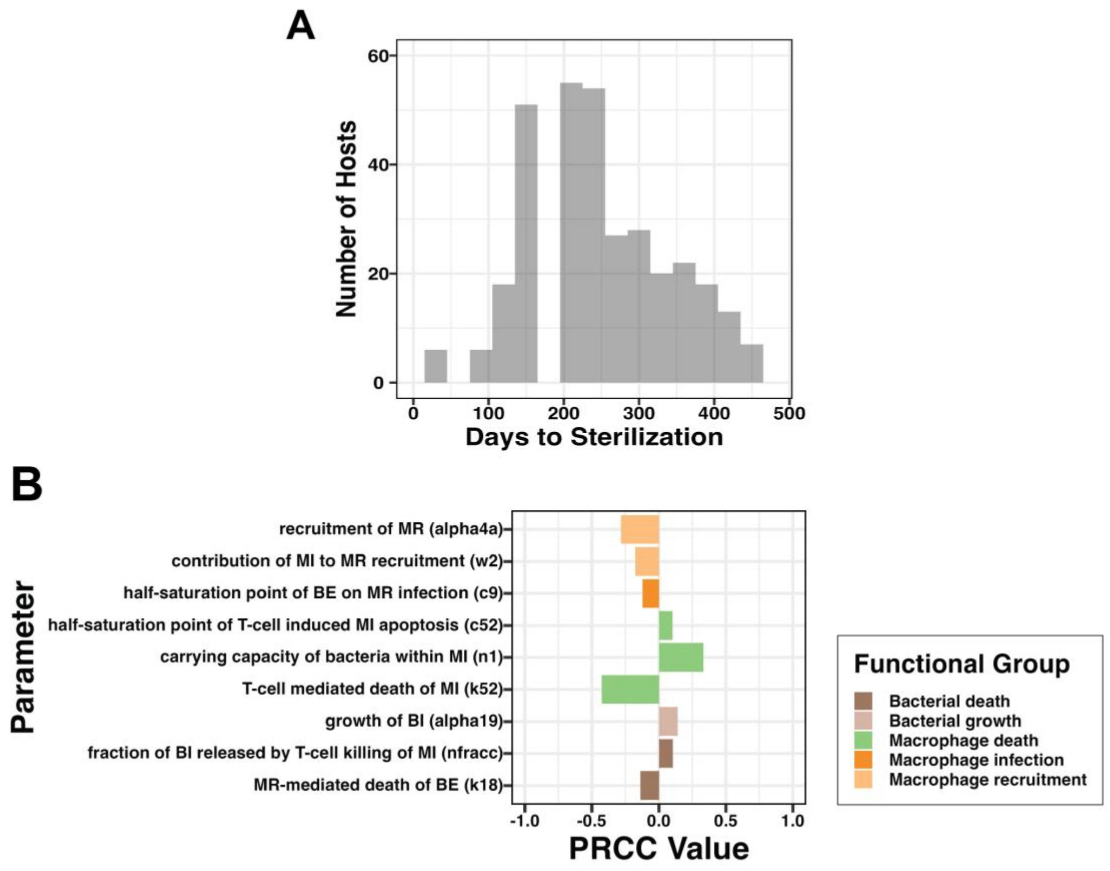

Fig A: ***Time-to-sterilization is driven by macrophage behavior within hosts with active pulmonary disease.*** Time to sterilization for a simulated LN is defined as the first-time post-LN-infection that a LN contains less than 0.5 total bacteria, or one day beyond the end of the simulation did not sterilize (see Methods). (A) Time to sterilization among 410 diseased LNs from 1000 simulated hosts that were sterilized within the 480-day simulation period. (B) Significant PRCC correlates between functional groups of parameters and output of interest, namely time-to-sterilization (significance with alpha = 0.01 after Bonferroni correction). Our analysis used 388 individual diseased LNs with granulomas from 1000 simulated hosts. Complete model state descriptions (MR, MI, E4, etc.) can be found in **Table 2** and parameter values in **Tables A, B, and C in S2 Appendix**.

As in the LTBI case presented in the main paper, we see similar results for time-to-sterilization distribution for virtual hosts with an active pulmonary infection (**Fig A, panel A**). Additionally, similar results are found with sensitivity analysis of time-to-sterilization for host with active pulmonary infection (**Fig SA, panel B**). One key difference is a lack of significant correlations between granuloma-associated T-cell proliferation and time-to-sterilization. This likely follows from continued APC stimulation within infected LN leading to a decreased role for granuloma-associated T-cell stimulation **(Fig 3A-B).**

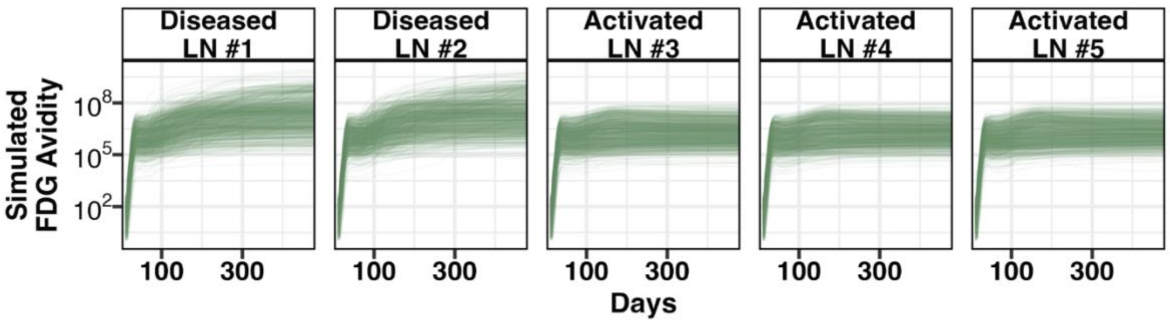

Fig B. P*redicted FDG avidity for hosts with active pulmonary disease.* Simulated FDG avidity of 5 simulated LNs over time post-infection starting at day 1.

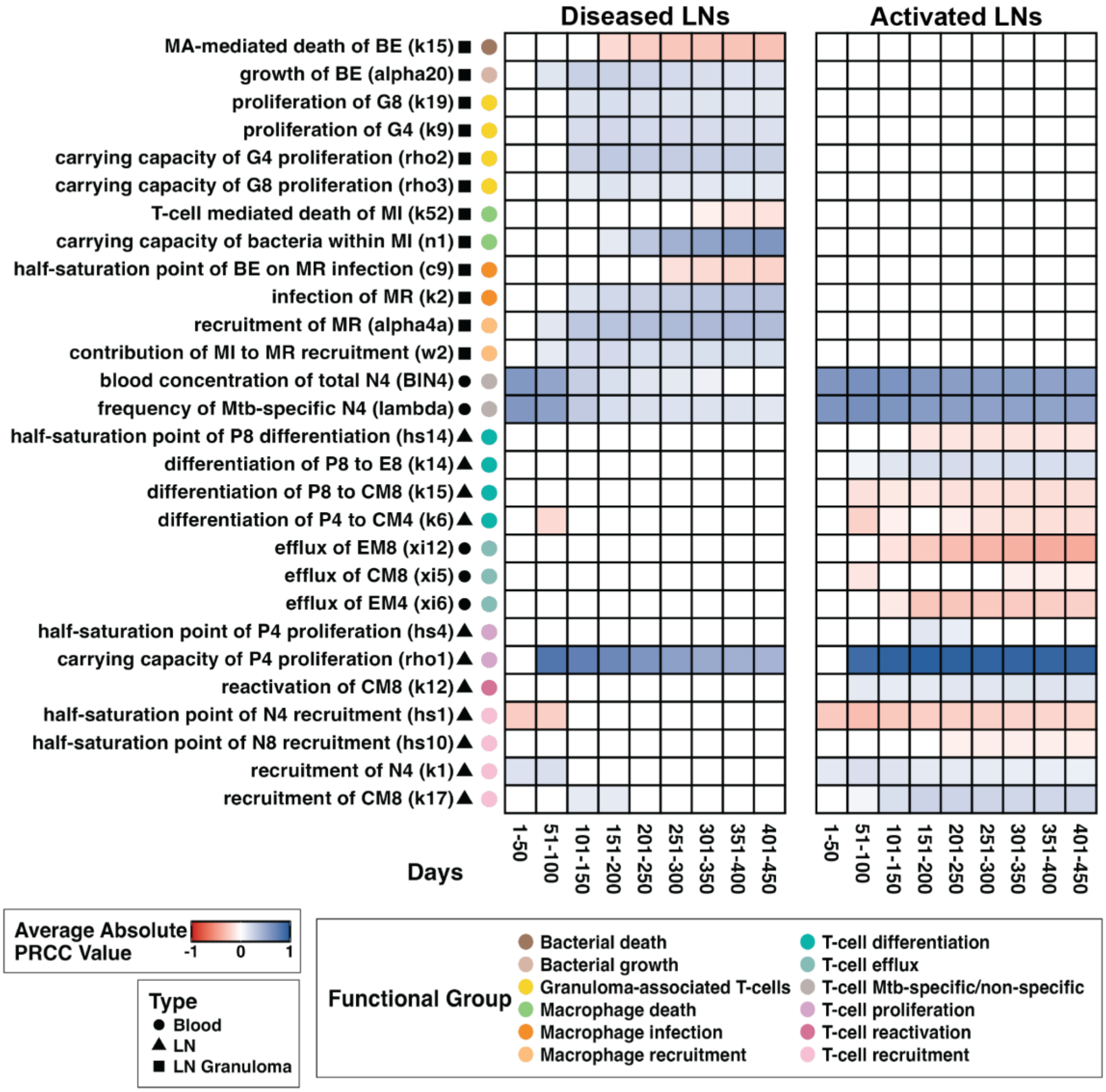

Fig C. ***Drivers of predicted FDG avidity within a host with active pulmonary disease.*** Left panel contains data from 2000 simulated individual diseased LNs and right panel contains data from 3000 individual simulated activated LNs with no granuloma forming. All simulated LNs are taken from the set of 1000 virtual hosts. Shading indicates correlation between parameter and FDG avidity during time interval *t* (given a parameter is at least significant for 30 days in *t*). White boxes indicate that the parameter is not significantly correlated with model outcome at any time points in *t*. A blue color indicates a positive correlation, and red color indicates a

negative correlation. Significance alpha = 0.01 after Bonferroni correction. Complete model state descriptions (MR, MI, E4, etc.) can be found in **Table 2**, and parameters in **Tables A, B, and C in S2 Appendix**.

For hosts with active pulmonary infection, we see very similar sensitivity analysis results of LTBI in main text. In hosts with active pulmonary infection, we find that parameters relating to the number of Mtb-specific T-cells in blood and LN have a weaker correlation (as compared to a host with LTBI). This suggests a continued high level of antigen presentation for hosts with active pulmonary infection leading to Mtb-specific T-cell numbers in the blood having a decreased role in determining simulated FDG avidity.

For hosts with active pulmonary infection, we find that there is a decrease in strength of correlation for differentiation of T cells into memory (rather than effector) T cells and simulated FDG avidity. This is driven by the APC profile (**Fig 3A-B**) leading to constant stimulation of the LN and the lack of opportunity (provided by decreased APC stimulation) for cells to become memory cells. Similarly, we see that carrying capacity for precursor cell proliferation has a stronger and longer correlation with simulated FDG avidity for hosts with active pulmonary infection. Precursor T-cell proliferation is driven by APCs. With persistence of high levels of APCs into late stages of our simulated infections, we expect the carrying capacity of precursor T-cell proliferation to be reached and its value to strongly drive the total number of cells in the LN (and in turn simulated FDG avidity) (**Fig 3A-B**).

At the end of our simulated infection for hosts with active pulmonary infection (481 days post infection), we see a slightly positive correlation between T-cell mediated death of macrophages and simulated FDG avidity. This correlation was not seen within LTBI hosts and suggests a larger role of T cells that emerges when APC stimulation is high.

Aside from these differences, sensitivity analysis findings for LTBI hosts and active pulmonary infections are similar. This lack of major differences between a host LN with active pulmonary infection versus a host with LTBI also suggests that LN granuloma-scale infection progression may be largely independent of pulmonary infection status.
