# Supplementary figures and images for: Distinguishing multiple roles of T cell and macrophage involvement in determining lymph node fates during Mycobacterium tuberculosis infection
Source: PLoS Comput Biol. 2025 May 7;21(5):e1013033. doi: 10.1371/journal.pcbi.1013033 (PMC12084042; doi:10.1371/journal.pcbi.1013033)

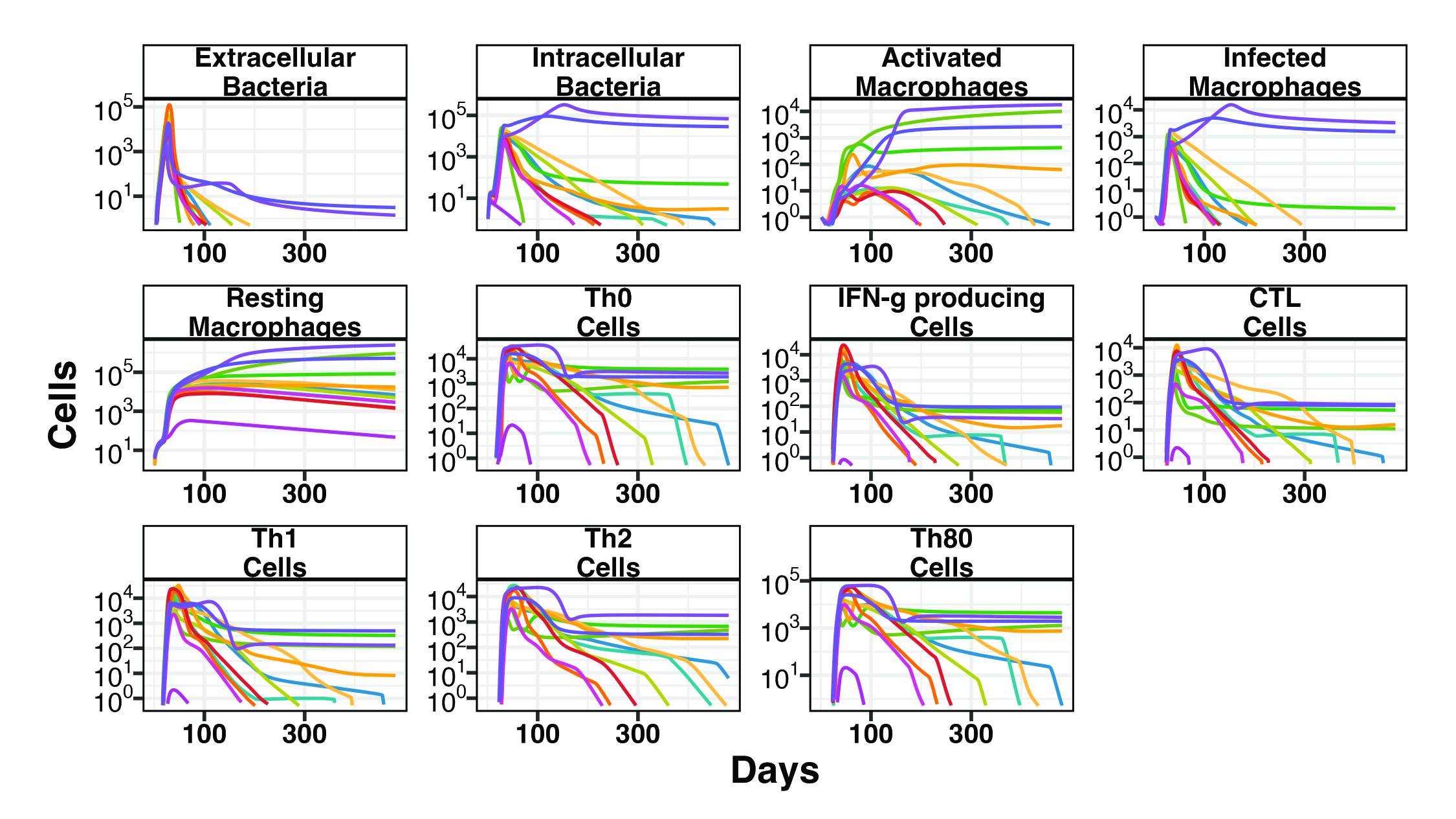

Supplement: S1 Fig — Model output for granulomas from a representative host that was used to generate APCs from a host with active pulmonary disease. Shown are cell numbers and bacterial levels for this representative active host (colors represent unique granuloma trajectories within our representative host). Two granulomas (above in purple) have high-burden, uncontrolled bacteria indicating active granulomas, and thus an active pulmonary infection. All other granulomas (other lines) are granulomas where bacteria are controlled or cleared. (TIF) [file pcbi.1013033.s004.tif]

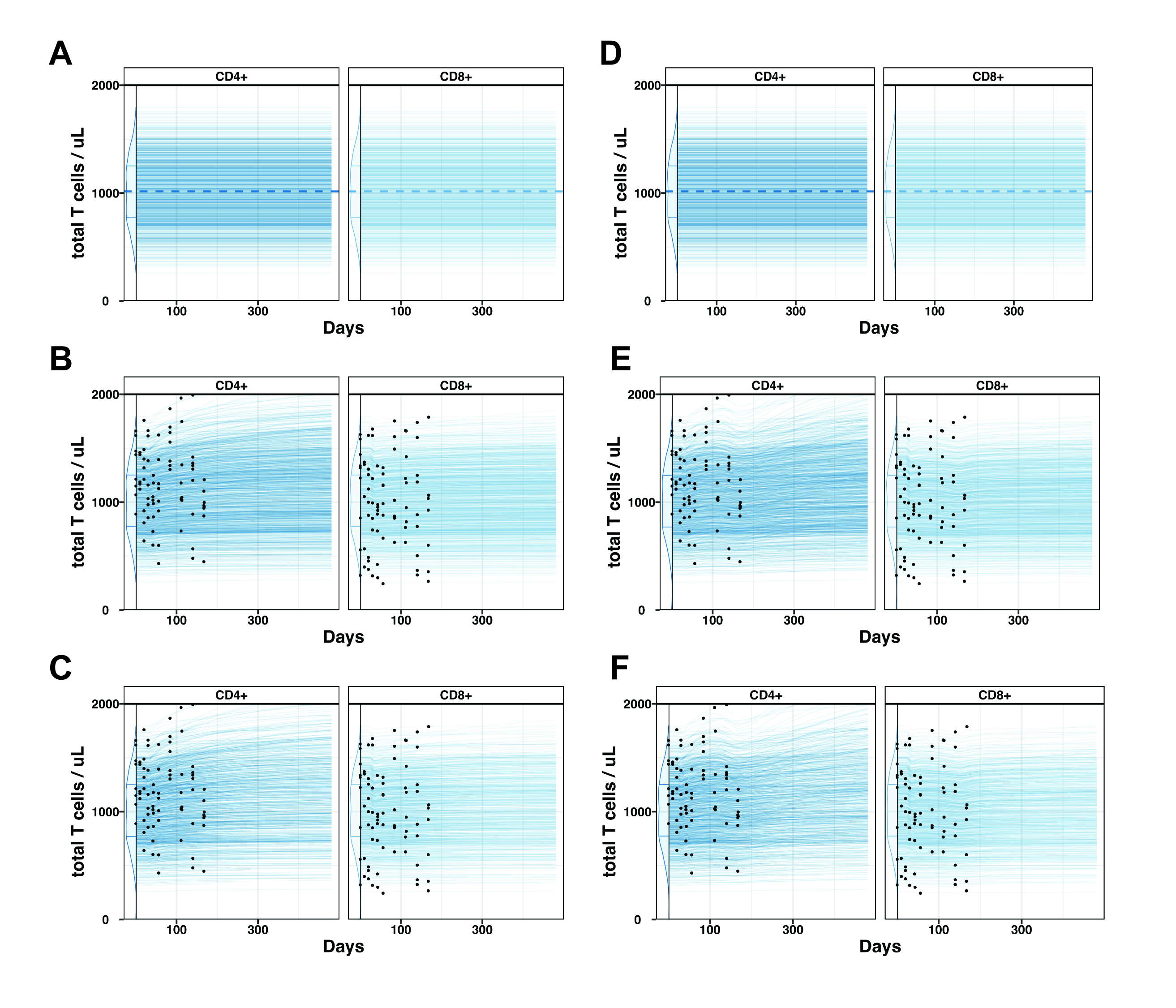

Supplement: S2 Fig — We simulated 1000 virtual hosts having both LTBI and active pulmonary disease (using the unique APC trajectories respectively). Our model is calibrated to capture key dynamics of total T cells in the blood within the uninfected (A, D), activated (B, E), and diseased (C, F) cases for virtual hosts with LTBI (A, B, C) and virtual hosts with active pulmonary infection (D, E, F). Uninfected hosts have no Mtb infection and no APC driven activation in their LNs. Activated hosts have five LNs receiving Mtb activated APCs. Diseased hosts have five activated LNs receiving Mtb activated APCs and LN granulomas forming in LN #1 and #2. We simulate 1000 separate virtual hosts for each case. Black dashed line in A and B represents average concentration of CD4 + and CD8 + T-cells in blood of a healthy animal. Flow cytometry data from individual NHPs is represented by black dots from [13] in B, C, E, and F. (TIF) [file pcbi.1013033.s005.tif]

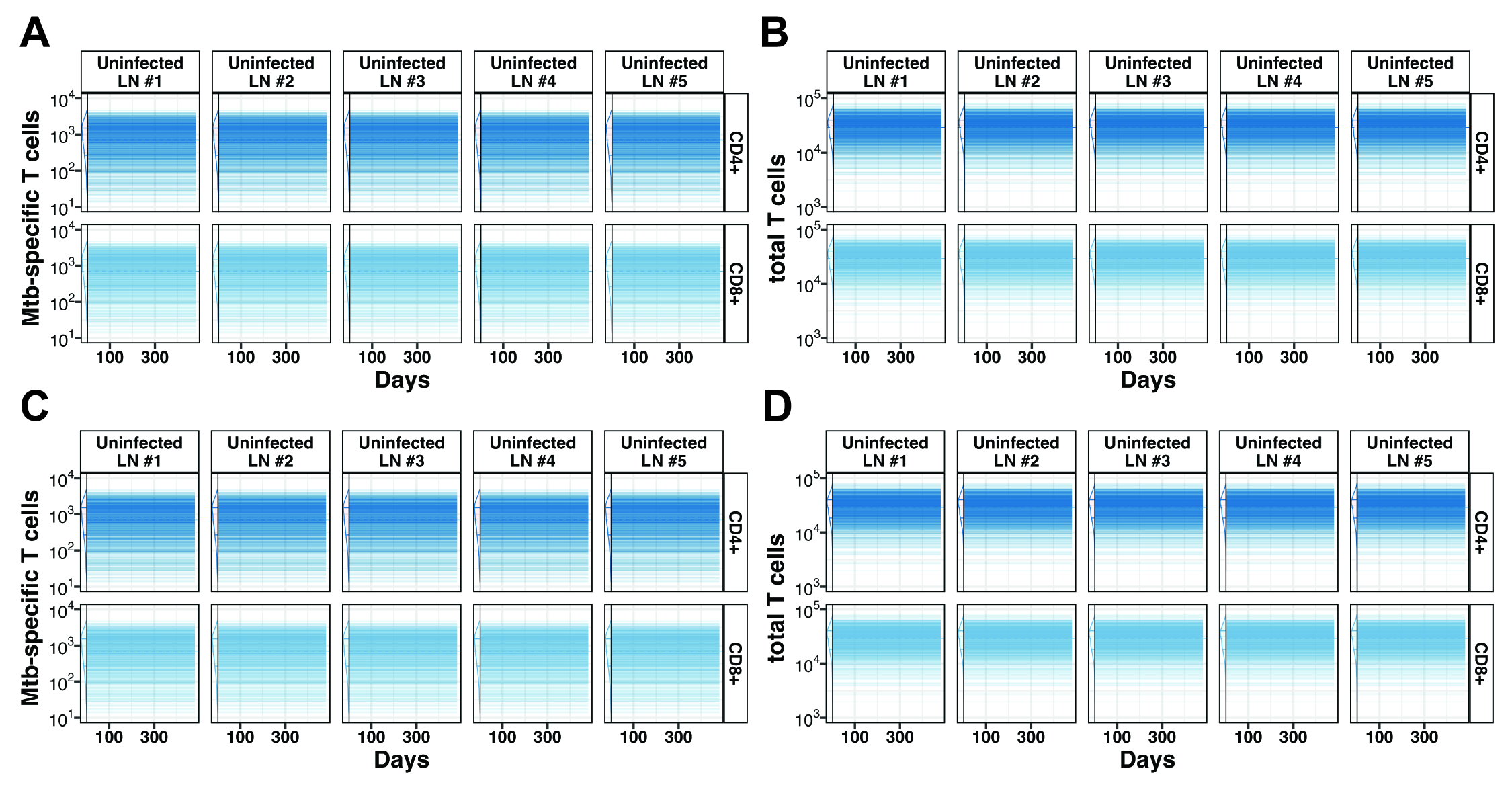

Supplement: S3 Fig — Our model is calibrated to capture key dynamics of Mtb-specific T cells (A, C) and total T cells (B, D) for virtual uninfected hosts with both LTBI (A, B) and active pulmonary infection (C, D). Uninfected hosts have no Mtb infection and no APC-driven activation in their LNs. We simulated 1000 separate virtual hosts for each case. In each plot, 1000 hosts are represented, each host LN is a line. (TIF) [file pcbi.1013033.s006.tif]

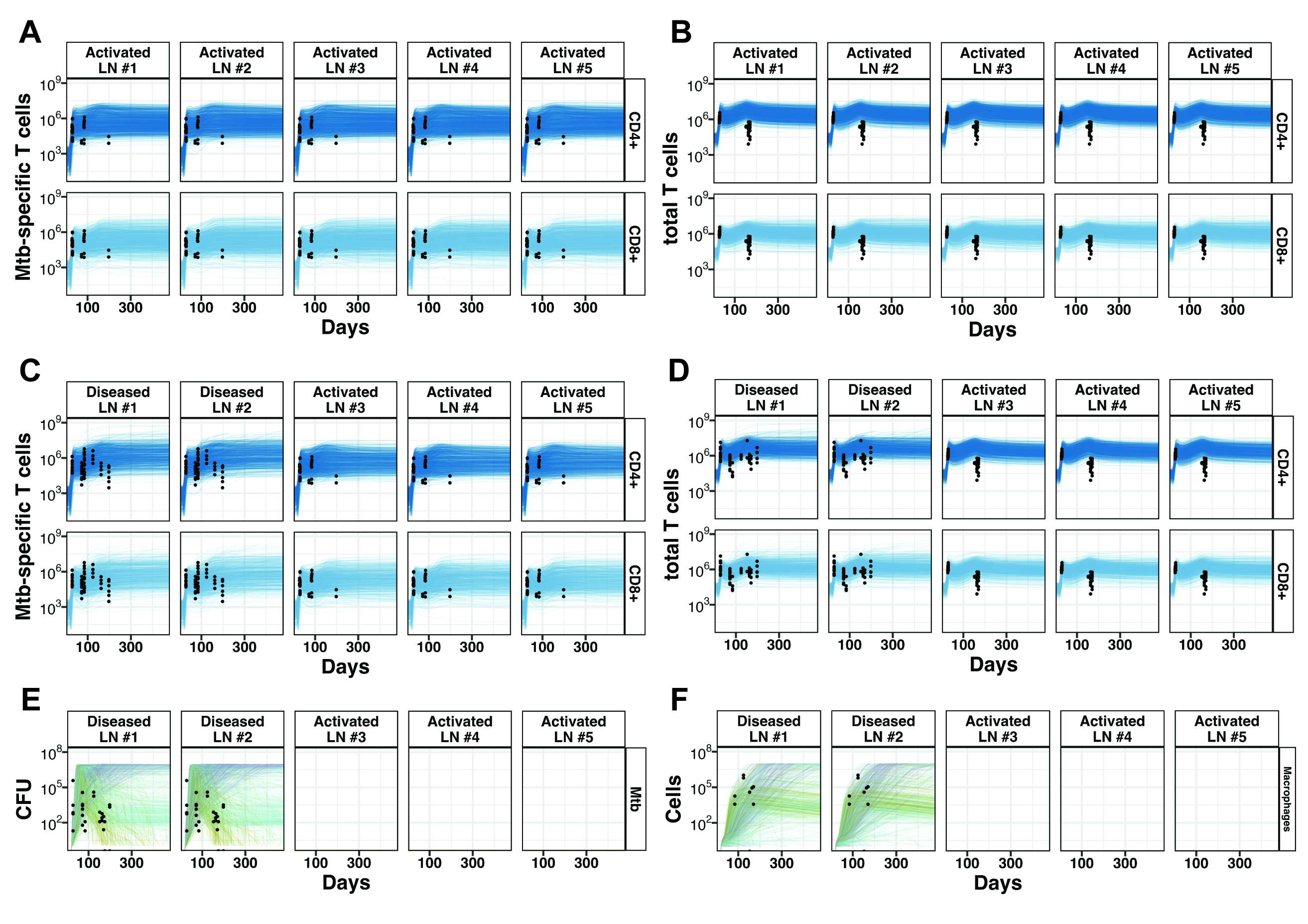

Supplement: S4 Fig — Our model is calibrated to capture key dynamics of Mtb-specific T cells (A, C) and total T cells (B, D) within activated (A, B) and diseased (C, D) cases. Activated hosts have five LNs receiving Mtb activated APCs. Diseased hosts have five activated LNs receiving Mtb activated APCs and LN granulomas forming in LN #1 and #2. For diseased LNs, our model captures the dynamics of LN bacterial load (E) and macrophages (F). We simulated 1000 separate virtual hosts for each case, generating a distinct trajectory for each of their LNs based on their parameterization. Lines in each plot show cell populations from the indicated LN within one host. For LN bacterial load (E) and macrophages (F), lines are colored by bacterial load trajectory: growing large (purple lines), stabilization (teal lines), and sterilization (yellow lines). Flow cytometry data from individual NHP LNs taken at necropsy are represented by black dots from [13]. Note that lines are truncated on virtual host death (see Methods, Section 6). (TIF) [file pcbi.1013033.s007.tif]

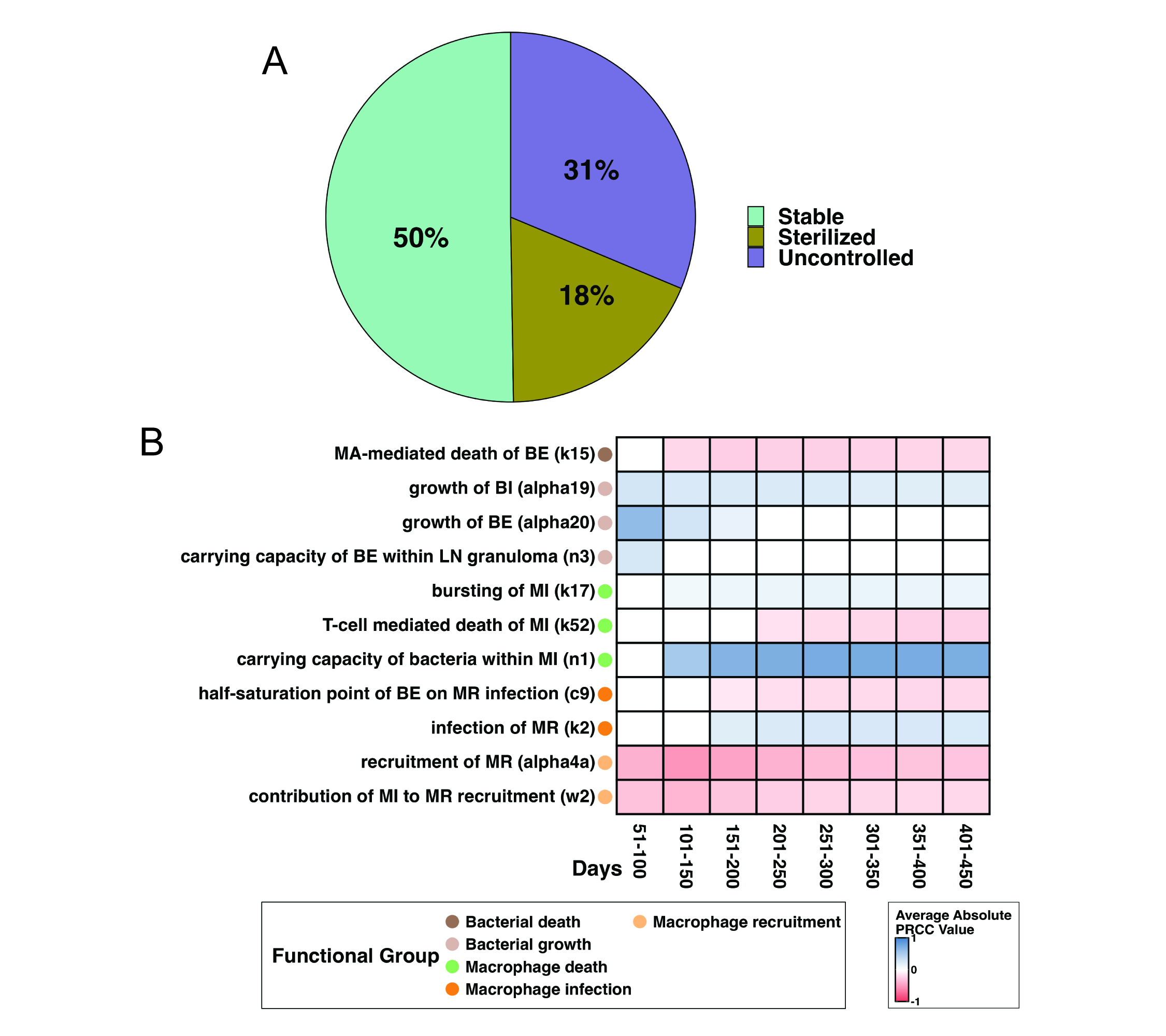

Supplement: S5 Fig — (A) Proportion of 2000 virtual LN granulomas by fate: no bacteria present (sterilized), stable bacterial growth (stable), and uncontrolled bacterial growth at 481 days post lung infection (N = 2000). (B) Summary of sensitivity analysis detailing significant parameters driving total bacterial load. PRCCs are binned into 50-day bins for ease of analysis (see Methods). Shading indicates average PRCC value during a time interval t (given a parameter is at least significant for 30 days in t). A blue color indicates a positive correlation, and red color indicates a negative correlation. Significance alpha = 0.01 after Bonferroni correction. Complete model state descriptions (MR, MI, E4, etc.) can be found in Table 2 in Methods and parameter value description found in Tables A, B, and C in S2 Appendix. (TIF) [file pcbi.1013033.s008.tif]
